# Microcircuit Rules Governing Impact of Single Interneurons on Purkinje Cell Output *In Vivo*

**DOI:** 10.1016/j.celrep.2020.02.009

**Published:** 2020-03-03

**Authors:** Charlotte Arlt, Michael Häusser

**Affiliations:** 1 Wolfson Institute for Biomedical Research and Department of Neuroscience, Physiology and Pharmacology, University College London, Gower Street, London WC1E 6BT, UK

**Keywords:** cerebellum, Purkinje cell, interneuron, patch clamp, inhibition, climbing fiber, glutamate spillover, in vivo, two-photon imaging, synaptic integration

## Abstract

The functional impact of single interneurons on neuronal output *in vivo* and how interneurons are recruited by physiological activity patterns remain poorly understood. In the cerebellar cortex, molecular layer interneurons and their targets, Purkinje cells, receive excitatory inputs from granule cells and climbing fibers. Using dual patch-clamp recordings from interneurons and Purkinje cells *in vivo*, we probe the spatiotemporal interactions between these circuit elements. We show that single interneuron spikes can potently inhibit Purkinje cell output, depending on interneuron location. Climbing fiber input activates many interneurons via glutamate spillover but results in inhibition of those interneurons that inhibit the same Purkinje cell receiving the climbing fiber input, forming a disinhibitory motif. These interneuron circuits are engaged during sensory processing, creating diverse pathway-specific response functions. These findings demonstrate how the powerful effect of single interneurons on Purkinje cell output can be sculpted by various interneuron circuit motifs to diversify cerebellar computations.

## Highlights

• Single spikes in single interneurons can inhibit Purkinje cell firing *in vivo*
• Climbing fiber input excites or inhibits interneurons via glutamate spillover *in vivo*
• Interneuron circuit motifs are spatially organized by depth in the molecular layer
• These direct and disinhibitory motifs can explain the responses to sensory stimulation

## Graphical Abstract

**Figure:**
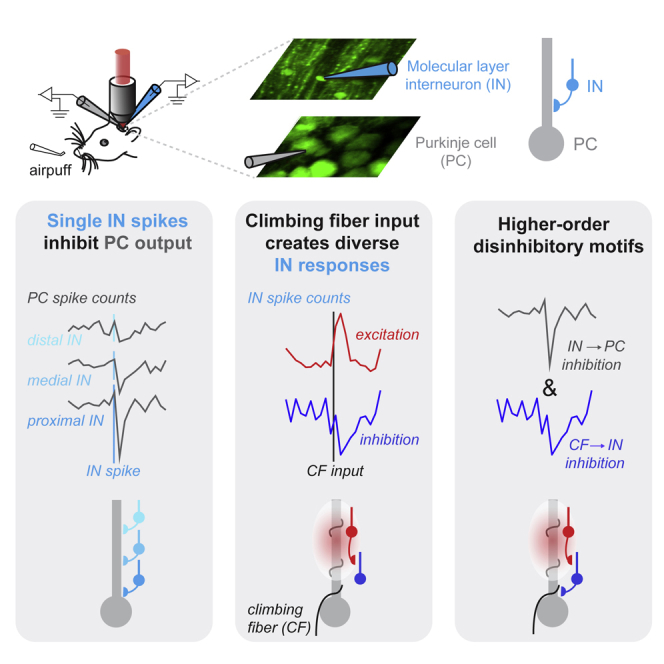

## Teaser

Using dual two-photon guided patch-clamp recordings from molecular-layer interneurons and Purkinje cells *in vivo*, Arlt and Häusser show that single-interneuron spikes can potently inhibit Purkinje cell output and uncover the microcircuit logic by which interneurons and their targets are recruited by different excitatory afferents.

## Introduction

Patterns of activity in neural circuits depend on the precise interplay of synaptic excitation and inhibition. Identifying how the activity of individual interneurons (INs) sculpts principal cell firing, and uncovering the logic by which incoming excitation engages inhibitory neurons, is crucial for understanding local computations and circuit dynamics (Isaacson and Scanziani, 2011, Tremblay et al., 2016). However, the impact of single INs on their targets *in vivo* is difficult to establish. Although numerous *in vitro* paired-recording studies have measured the consequences of single IN spiking on postsynaptic output, in both cortical (Cobb et al., 1995, Miles et al., 1996) and cerebellar (Häusser and Clark, 1997, Oldfield et al., 2010) circuits, differences in the levels of activity (Bernander et al., 1991, Destexhe and Paré, 1999, Rapp et al., 1992, Waters and Helmchen, 2006), extracellular calcium levels (Borst, 2010), and neuromodulatory states present *in vivo* make it challenging to extrapolate from slice studies to the *in vivo* situation. Moreover, the slicing procedure may truncate or modify axonal and dendritic morphology (Jiang et al., 2015, Kirov et al., 1999), thereby changing the connectivity patterns and the efficacy of monosynaptic connections. Measuring the functional impact of single INs *in vivo* is therefore essential if we are to understand how inhibitory networks exert their effects.

Measuring the impact of monosynaptic connections is challenging *in vivo*, and no reports exist of dual patch-clamp recordings of single INs and synaptically connected postsynaptic cells in the mammalian brain. Paired extracellular recordings have reported inhibitory correlations between IN spikes and postsynaptic spikes (Barthó et al., 2004, Blot et al., 2016, Diba et al., 2014), but the interpretation of such experiments is complicated by synchrony and common input, as well as the difficulty of unambiguously identifying INs from extracellular recordings. That single INs can affect the local network *in vivo* has been suggested by experiments in which activating single cortical INs with spike trains can produce a behavioral report in rodents (Doron et al., 2014). However, how these INs exert their behavioral effects via network activity has not been established, and the impact of single IN spikes has not been measured.

In the cerebellar cortex, extrapolating from *in vitro* recordings is further hindered by recent findings revealing that the cerebellar inhibitory network exhibits unexpected complexity. The molecular layer IN population is morphologically (Eccles et al., 1967, Palay and Chan-Palay, 1974, Sultan and Bower, 1998) and functionally (Blot and Barbour, 2014, Pouzat and Hestrin, 1997) diverse. INs inhibit one another (Häusser and Clark, 1997, Kondo and Marty, 1998, Mittmann et al., 2005, Rieubland et al., 2014), and the IN network shows structured connectivity patterns organized in the sagittal plane (Rieubland et al., 2014). INs are highly sensitive to excitatory input (Carter and Regehr, 2002) and can be excited by inputs from single granule cells (Barbour, 1993), which in turn excite Purkinje cells (PCs), forming a feedforward inhibition motif (Mittmann et al., 2005). Furthermore, climbing fiber (CF) input to PCs has been shown to excite INs via an unconventional spillover pathway (Szapiro and Barbour, 2007), which can also exhibit disynaptic inhibition (Coddington et al., 2013, Mathews et al., 2012). Thus, predicting how single INs exert their influence over PCs *in vivo*, particularly during sensory processing, is not trivial and requires direct measurement.

Here we have performed dual targeted patch-clamp recordings from INs and PCs to directly measure the influence of single INs on PC spiking *in vivo*. Our data reveal differential recruitment of functional IN-PC connectivity by different excitatory pathways, with important implications for understanding the functional role of inhibition in the cerebellar cortical network.

## Results

### Single IN Spikes Inhibit Purkinje Cell Firing *In Vivo*

**Figure 1:**
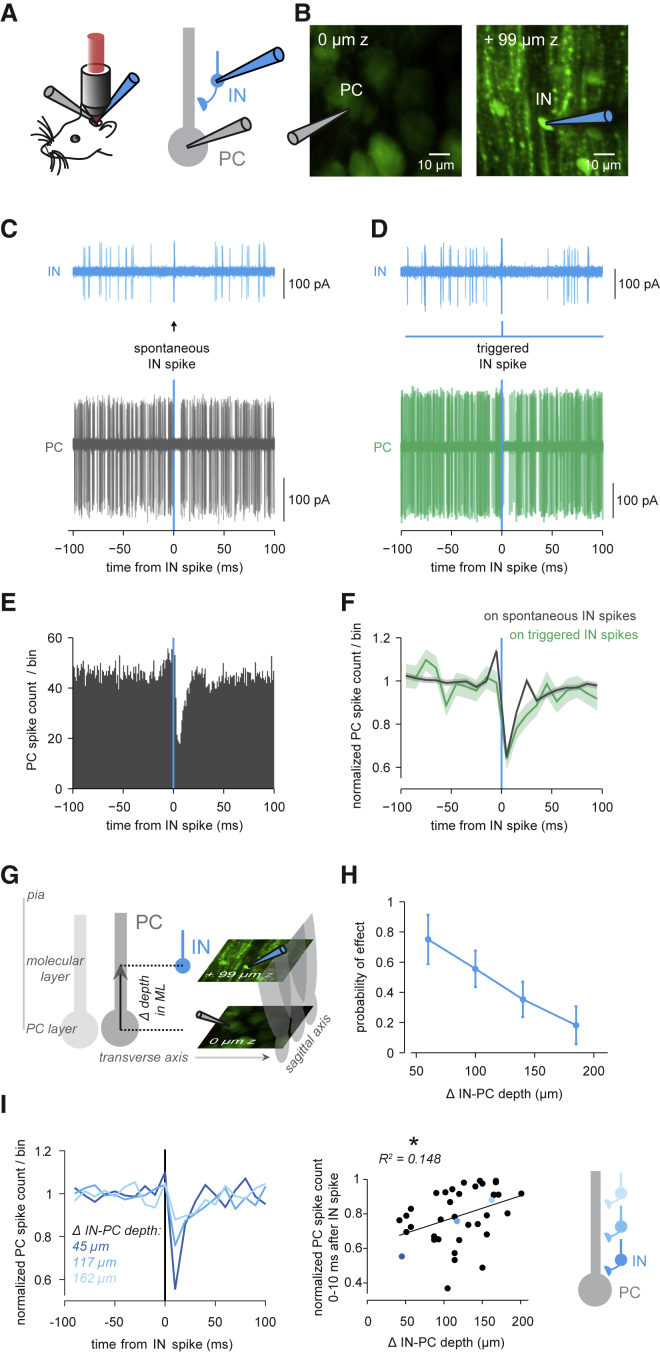
Single IN Spikes Inhibit PC Spiking *In Vivo* (A) Left: dual two-photon targeted patch-clamp recordings from INs and PCs *in vivo*. Right: schematic of paired recording configuration. (B) Two-photon average intensity projections showing a dual PC (left) and IN (right) cell-attached recording configuration. (C) IN trace triggered on 20 consecutive spontaneous IN spikes, traces overlaid; below, corresponding overlaid traces of simultaneous PC recording (complex spikes removed). IN spikes before or after time point 0 ms were used to align PC activity in previous or consecutive trials, respectively. PC pauses were not present after IN spikes before or after the 0 ms time point because previous or following IN spikes were irregularly timed. (D) IN spikes directly triggered by a brief (0.2 ms) voltage pulse via the patch pipette. Below: corresponding overlaid PC traces. (E) PC simple spike histogram aligned to spontaneous IN spikes (bin size = 1 ms). (F) Normalized PC spike probability/bin aligned to spontaneous (gray) and triggered (green) IN spikes (bin size = 10 ms). Shadings denote ±SEM. (B)–(F) are from the same dual recording. (G) Illustration of intersomatic Euclidean IN-PC distance in the transverse plane and sagittal plane and as the Δ depth in the molecular layer (ML). The center of the PC soma denotes a 0 μm distance. (H) Fraction of pairs with significant (PC *Z* score < −3) IN-PC inhibition across ML depth IN-PC distance. Error bars show SD based on bootstrap analysis. (I) Left: baseline-normalized PC spike counts/bin for 3 example pairs with the IN soma positioned deep (dark blue), intermediate (blue), and superficial (light blue) in ML. Middle: for IN-PC pairs within the 30 μm intersomatic transverse distance, the ML depth IN-PC distance is plotted versus the normalized PC spike count in the 0–10 ms bin after the IN spike. The black line indicates the linear regression line, p = 0.017. Colors indicate the pairs shown on the left. Right: schematic of IN-PC inhibition graded by IN depth in ML.

We performed simultaneous dual targeted patch-clamp recordings from INs and PCs in vermis and crus II of isoflurane-anesthetized parvalbumin-positive (PV^+^)-GFP mice (Figures 1A and 1B). INs and PCs were clearly distinguishable based on the location and size of their somata, allowing us to target PCs and neighboring INs in the molecular layer. Both INs and PCs were spontaneously active (Armstrong and Rawson, 1979, Chu et al., 2012, Eccles et al., 1966), with INs spiking at 8.0 ± 7.7 Hz and PCs at 26.4 ± 15.5 Hz (n = 56 paired cell-attached recordings, mean ± SD). PCs also exhibited complex spikes at 1.2 ± 0.6 Hz. We first used cross-correlograms between spontaneous IN and PC spikes to assess functional IN-PC interactions. Single IN action potentials were correlated with a powerful transient inhibition of PC simple spikes (Figures 1C and 1E). Of 56 IN-PC pairs, 24 showed significant PC inhibition (i.e., *Z* scores < −3 in the 0–10 ms bin from the IN spike). In those pairs, on average, the PC spiking probability dropped by ∼30% (average baseline-normalized PC spike probability of 0.71 ± 0.14 in the 0–10 ms bin after an IN spike). We also quantified PC inhibition as the average number of dropped PC spikes per IN spike, i.e., the net spike change (Figure S1). In the 24 pairs with significant IN-PC inhibition, on average, each IN spike produced a net change of −0.12 ± 0.11 spikes in the PC (Figure S1E), whereas this measure was close to zero in the remaining pairs without significant IN-PC inhibition (−0.03 ± 0.18 net spike change, n = 32, p < 0.005 comparing net spike changes in the two groups).

To examine the dynamics of the connection, we compared inhibition mediated by short (<20 ms) or long (>100 ms) IN inter-spike intervals (ISIs) (Figures S2A and S2B). When IN spikes occurred in doublets (IN ISIs < 20 ms), the second spike produced a comparable decrease in PC spike probability (normalized PC spike count/bin of 0.79 ± 0.42 and 0.76 ± 0.39 in bins 0–10 and 20–30 following the first IN spike, respectively; p = 0.4, n = 11 pairs showing significant IN-PC inhibition) (Figure S2C), comparable to paired-pulse dynamics at the mature IN-PC synapse (Pouzat and Hestrin, 1997). In contrast, when aligning PC spikes to IN spikes with large ISIs, following the initial PC spike probability decrease right after the IN spike, PC spiking probability slowly rose to levels above baseline, suggesting disinhibition of PC spikes as a result of IN spike pauses (1.09 ± 0.08 in a 50–100 ms window post-IN spike compared with pre-spike baseline, p < 0.001, n = 11 pairs) (Figure S2C).

Next, we directly stimulated IN spikes via the cell-attached recording pipette (Figure 1D; Barbour and Isope, 2000) to provide a causal assay of IN-PC inhibition. This method of single spike triggering was specific to the stimulated IN in most trials (Figure S3; see also van Welie et al., 2016). Triggering a single spike in a single IN produced a clear decrease in PC spiking probability, comparable to the effect of spontaneous IN spikes at the same connection (Figures 1D and 1F). Pairs in which the triggered IN spike resulted in significant PC inhibition (pairs with a PC *Z* score < −3 in the 0–10 ms bin after the triggered IN spike, n = 6/30 pairs) also exhibited significant PC inhibition associated with spontaneous IN spikes. Furthermore, the baseline-normalized PC spike probabilities after triggered and spontaneous IN spikes were highly correlated (n = 30, p = 0.024) (Figure S4A), and the values corresponding to the 6 pairs with significant PC inhibition after triggered IN spikes were close to the unity line, consistent with a strong correlation between spontaneous and triggered IN-PC effects. Finally, we examined the effect of a triggered IN spike on the succeeding ISI in the postsynaptic PC in pairs with significant PC spike probability decreases. PC ISIs were significantly delayed, by ∼5 ms on average, by single IN spikes (Figures S4B–S4D). Altogether, these results indicate that single spikes in single INs can exert a powerful influence on the spiking of postsynaptic PCs *in vivo*. They also confirm that functional connectivity between INs and PCs can be read out using cross-correlation analysis of spontaneous spikes.

The efficacy of IN-PC inhibition depended on IN location in the molecular layer: IN-PC inhibition was more prevalent in deep INs than in superficial INs (Figure 1H) (mean distance to PC: 106.3 ± 43.9 μm, n = 24, with significant PC inhibition versus 135.8 ± 30.6 μm, n = 30, without significant PC inhibition, p = 0.006). The strength of PC inhibition, measured as the baseline-normalized PC spike probability following the IN spike, was inversely related to increasing IN-PC distance in the molecular layer (Figure 1I): the deeper the IN soma, the bigger the inhibitory IN-PC effect (R^2^ = 0.148, p = 0.017, IN-PC pairs within a 30 μm transverse distance only). Similar findings were made with directly triggered IN-PC inhibition (Figures S6C and S6D). Finally, IN-PC pairs with significant PC inhibition were closer in transverse distance than pairs without inhibition (11.7 ± 7.6 μm, n = 24, versus 20.1 ± 17.0 μm, n = 30, p = 0.010) (Figures 1G, S5, and S6), while there was no relationship with location in the sagittal plane (Figure S6B). Altogether, these results confirm that IN-PC inhibition is restricted to the sagittal plane and can occur at relatively large distances within that plane (Figures S6A and S6B), in line with IN axons extending hundreds of micrometers sagittally (Sultan and Bower, 1998). Our data thus indicate that proximal basket cells are more potent than superficial stellate cells in inhibiting PC spiking but do not support a binary IN categorization based on IN depth in the molecular layer, suggesting a continuum of inhibition instead (Figure 1I).

### IN-IN Cross-Correlograms Are Diverse, with Directional Inhibition from Top to Bottom in the Molecular Layer

**Figure 2:**
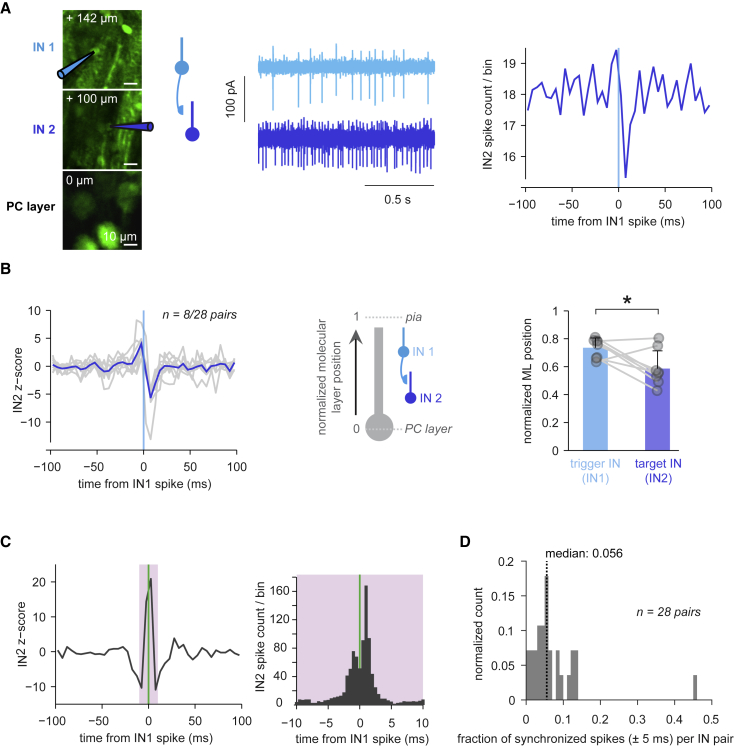
Interneurons Inhibit Each Other with a Top to Bottom Organization in the Molecular Layer (A) Left: two-photon average intensity projection showing a dual IN-IN cell-attached recording. Middle: spontaneous activity of the IN-IN pair. Right: average IN2 spike count/bin aligned to IN1 spikes (bin size = 5 ms). (B) Cross-correlograms of 8 IN-IN pairs with significant unidirectional spike probability decreases, dark blue: average. The trigger, presumed to be presynaptic IN, was denoted as IN1, and the target was denoted as IN2. Middle: schematic illustrating the measurement of IN-IN distance in the molecular layer (ML) as normalized ML positions. Right: ML positions of trigger INs and target INs (mean + SD, p = 0.02, Wilcoxon signed-rank test). (C) Left: cross-correlogram of another IN-IN pair (5 ms bin size) exhibiting precise synchrony. Right: higher temporal resolution (bin size = 0.5 ms) version of cross-correlogram on left, showing two peaks at ±1 ms lag. Purple shading indicates the temporal window shown at a higher resolution. (D) Across all IN-IN pairs, distribution of fraction of synchronized spikes (spikes within ±5 ms of the spike in the other IN) per IN pair. The dotted black line shows the distribution median.

To characterize interactions within the IN population, we performed dual targeted patch-clamp recordings from IN pairs (Figures 2A and S7A). A sizable fraction of IN-IN cross-correlograms (8/28 pairs) exhibited short-latency spike probability decreases in one IN after spikes in the other (Figure 2A), thus likely reflecting monosynaptic γ-aminobutyric acid (GABA)-ergic inhibition. In these pairs, a single IN spike resulted in the spiking probability being transiently reduced by ∼20% in the other IN (average baseline-normalized IN spike probability of 0.81 ± 0.16 in the 0–10 ms bin after an IN spike). Next, we categorized INs as trigger (presynaptic) and target (postsynaptic, i.e., inhibited) INs and measured their somatic depth in the molecular layer. We found that trigger INs tended to be more superficial than their targets (Figure 2B) (mean normalized molecular-layer positions of 0.74 ± 0.07 versus 0.59 ± 0.13, n = 8, p = 0.02, 0 denotes the PC layer). These results demonstrate that *in vivo*, INs can significantly inhibit one another, and this inhibitory connectivity is organized from top to bottom in the molecular layer (Rieubland et al., 2014). IN-IN cross-correlograms were more diverse than IN-PC cross-correlograms (Figure S7B); in addition to unidirectional inhibitory connectivity, we observed co-activation without unilateral inhibition in 6/28 pairs (example in Figure S7C, left), demonstrating some degree of synchronization of the IN population. The hallmark of precise synchrony mediated by electrical coupling (Mann-Metzer and Yarom, 1999, Rieubland et al., 2014) is symmetric peaks at ±1 ms lag in the cross-correlogram (van Welie et al., 2016). Such a pattern was only observed in one of our IN-IN pairs (Figure 2C), suggesting that in most cases, loose synchrony results from common input rather than strong gap-junction coupling. Accordingly, across our IN-IN recordings, the fraction of synchronized spikes per pair (i.e., spikes of one IN within ±5 ms of spikes of the other IN) was relatively low (median of 5%) (Figure 2D). Thus, except for rare cases of strong gap-junction coupling, most spontaneous IN spikes were not accompanied by highly synchronous spikes in other INs. This is consistent with the similarity between spontaneous and triggered IN-PC inhibition and corroborates our conclusion that single spikes in single INs can potently inhibit downstream PCs.

### Co-activation of IN-PC Pairs Depends on IN-PC Inhibition

In some IN-PC recordings, PC spike probability significantly increased in the −10 to 0 ms bin preceding the IN spike (Figure S8A). Interestingly, and in contrast to our dual IN recordings, in which co-activation could occur without inhibition, this increase was only observed in IN-PC pairs that also showed significant IN-PC inhibition (Figure S8); in other words, the occurrences of co-activation and inhibition were not independent of each other (Fisher’s exact test, p = 0.004) (Figure S8B). Previous work suggested that this co-activation peak preceding IN-PC inhibition is driven by shared granule-cell input (Blot et al., 2016), such that when co-activation and inhibition are both observed in single pairs, this indicates a feedforward inhibition motif. If shared input fully accounted for the co-activation peak, one would expect the timing of increased spiking would be temporally restricted to a relatively narrow window of ∼±10 ms, a timescale at which shared inputs are known to synchronize neuronal spiking (Ostojic et al., 2009). In our case, increased PC spiking would be restricted only to negative lags from the IN spike, i.e., −10 to 0 ms, because the positive lag is the time window of IN-PC inhibition. Conversely, elevated PC spiking probability before the IN spike can be explained by an absence of IN spikes in that time window, resulting in PC disinhibition. If that were the case, the temporal window of PC disinhibition should depend on the previous IN ISI. When aligning PC spikes to IN spikes depending on the previous IN ISI, the temporal profile of increases in PC spiking probability was tightly linked to the previous IN ISI, rising tens of milliseconds before the IN spike if aligned to long preceding ISIs (Figure S8C). This effect was apparent in pairs with strong IN-PC co-activation and in pairs with weaker co-activation *Z* scores (Figure S8C, left and middle). In both cases, the strength of IN-PC inhibition was similar. These results show that under spontaneous conditions, increases in PC spiking before IN spikes are tightly linked to IN-PC inhibition, and this effect may result not only from shared granule-cell input but also from PC disinhibition.

### CF Input Activates Many INs but Inhibits INs Deep in the Molecular Layer

**Figure 3:**
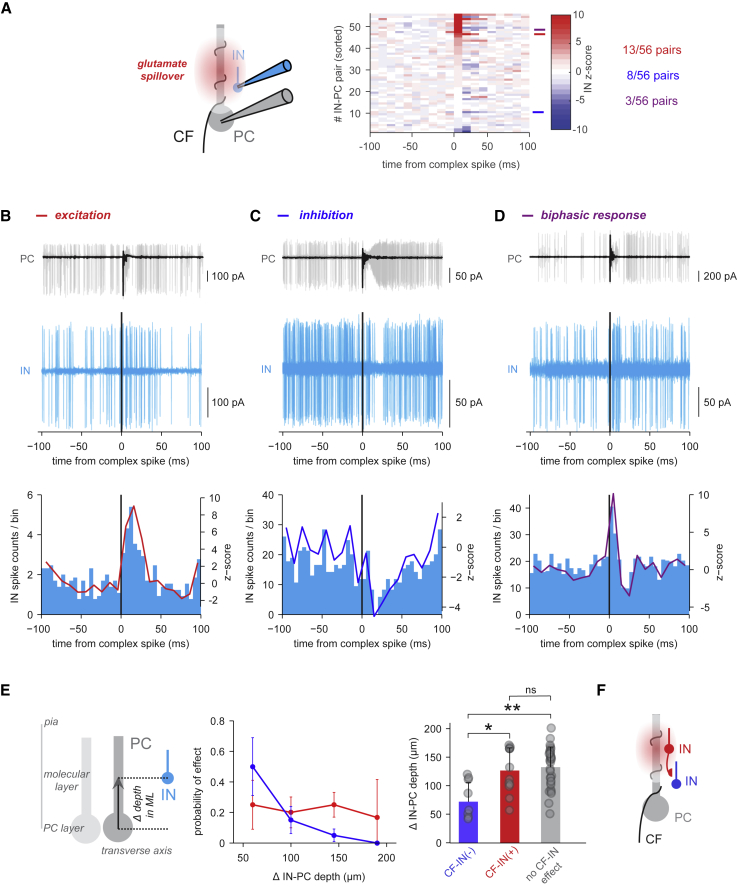
CF Input Activates Many INs but Inhibits INs Deeper in the Molecular Layer (A) Left: schematic of the recording configuration. Right: *Z* scores of IN spikes aligned to PC complex spikes (color axis clipped at *Z* score = ±10 for better visualization, bin size = 10 ms). Pairs were sorted by minimal *Z* score in 0–10 ms bin. (B) Top: raw overlaid PC traces aligned to 50 consecutive complex spikes. Overlays of raw traces always show a small subset of all traces for better illustration. The colored bar on the top left indicates the identity of the cross-correlogram in the overview in (A), right. Second row: corresponding overlaid IN traces aligned to 150 additional complex spikes. Third row: IN histogram aligned to complex spikes (bin size = 5 ms, left y axis), and *Z* scores of IN spike count/bin aligned to complex spikes (bin size = 10 ms, right y axis). (C) Analogous to (B), but for another recording. Overlaid PC and IN traces aligned to 76 consecutive complex spikes. (D) Another dual recording. Overlaid IN traces aligned to 60 consecutive complex spikes. (E) Left: schematic illustrating the measurement of the IN-PC depth distance in the molecular layer (ML). Prevalence of pure CF-IN excitation (red, n = 13) and pure CF-IN inhibition (blue, n = 8) across the IN-PC ML depth distance. Error bars show SD based on bootstrap analysis. Right: IN-PC depth distance in INs with CF-IN inhibition, INs with CF-IN excitation, and those with no significant CF-IN effect (mean + SD, p = 0.038 (CF-IN- versus CF-IN+), p = 0.008 (CF-IN- versus no CF-IN effect), p = 0.976 (CF-IN+ versus no CF-IN effect), Kruskal-Wallis plus multiple comparisons test). (F) Schematic illustrating CF-IN excitation and delayed CF-IN inhibition in a deeper IN via directional IN-IN inhibition.

We next addressed whether CF input can modulate IN spiking *in vivo*, using spontaneously occurring PC complex spikes as a readout of local CF input during simultaneous IN-PC recordings. We found IN excitation after CF input in more than 20% of our recordings: in 13 of 56 pairs, the IN exhibited a pure spike probability increase (Figure 3A). These excitatory responses started immediately after the complex spike but had a slow rise time, peaking at a latency of 10–20 ms (Figure 3B) (*Z* score > 3 from 10–20 ms after the complex spike), and are thus consistent with extrasynaptic glutamate spillover from CF-PC synapses (Mathews et al., 2012, Szapiro and Barbour, 2007). Another subgroup of INs exhibited purely inhibitory responses to CF activation (Figure 3C) (*Z* score < −3 from 10–30 ms postcomplex spike, n = 8). Inhibitory responses occurred later than the excitatory responses observed in other INs (*Z* score thresholds crossed at 4.2 ± 2.8 and 13.8 ± 6.4 ms for IN spike increases and decreases, respectively; p = 0.0006). The slower time course of inhibitory responses suggests that they result from CF input recruiting INs that in turn inhibit other INs outside of the glutamate spillover diffusion limit (Coddington et al., 2013). Finally, in 3 pairs, we found a combination of IN spike increases and decreases, with the increase always preceding the decrease (Figure 3D), presumably resulting from a combination of direct excitation by glutamate spillover followed by disynaptic inhibition.

Disynaptic inhibition mediated by CF-IN activation should be reflected in inhibition predominating in deeper INs, resulting from the top-to-bottom organization of molecular-layer IN connectivity (Rieubland et al., 2014; Figure 2B). Pure CF-IN inhibition was more prevalent in deeper INs, whereas pure CF-IN excitation did not depend on IN depth (Figures 3E and 3F). On average, INs purely inhibited following CF input were deeper than INs exhibiting excitation only (78.4 ± 33.3 μm, n = 8, versus 126.5 ± 39.8 μm, n = 12, p = 0.038) or INs without an effect of CF input (78.4 ± 33.3 μm, n = 8, versus 132.5 ± 35.2 μm, n = 31, p = 0.008, Kruskal-Wallis plus multiple comparisons test) (Figure 3D; see Figure S9 for CF-IN effects along transverse and sagittal distances). These results indicate that spontaneous CF activity both activates INs and disynaptically inhibits deeper INs *in vivo*.

### CF Input Inhibits INs with a Strong Impact on PC Spiking

**Figure 4:**
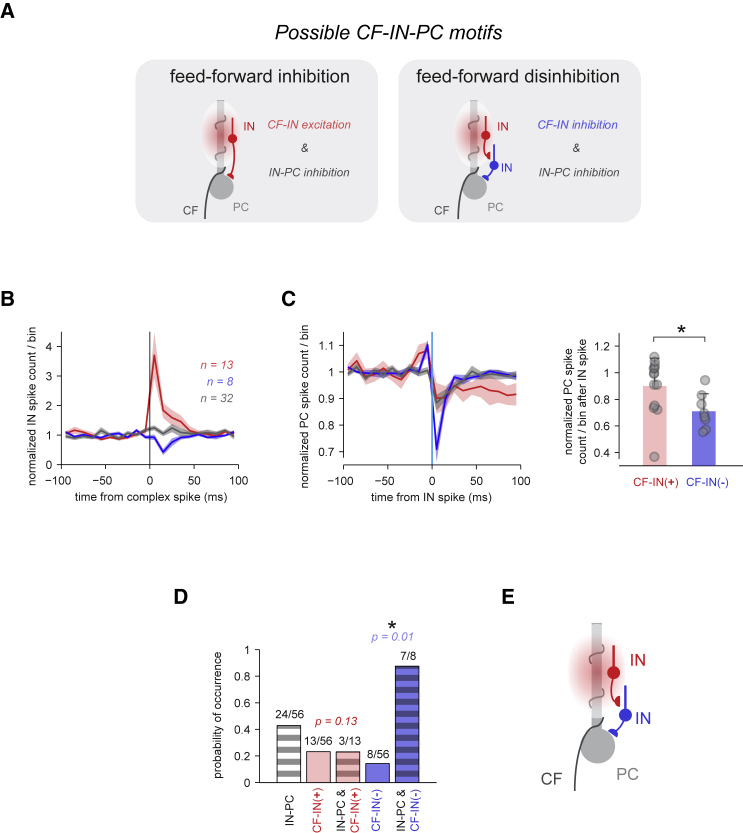
CF Input Inhibits Those INs with an Impact on PC Spiking (A) Schematics of possible CF-IN-PC motifs. Left: CF-IN-PC feed-forward inhibition circuit; i.e., CF-IN excitation (CF-IN+) co-occurring with IN-PC inhibition in the same IN-PC pairs. Right: schematic of a CF-IN-PC feed-forward disinhibition motif; i.e., CF-IN inhibition (CF-IN−) co-occurring with IN-PC inhibition. (B) Normalized mean IN spike counts/bin aligned to complex spikes. Pairs are grouped based on IN responses to CF input: significant excitation (red), inhibition (blue), or no response (gray). (C) Left: normalized PC spike counts/bin aligned to IN spikes, same grouping as in (B). Shadings denote ±SEM. Right: normalized PC spike count in the 0–10 ms bin after the IN spike in CF-IN− pairs and CF-IN+ pairs (mean + SD, p = 0.018, Wilcoxon rank-sum test). (D) Absolute probabilities of observing significant IN-PC inhibition (gray stripes), CF-IN+ (red), and CF-IN− (blue), and conditional probabilities of observing IN-PC inhibition given CF-IN+ or CF-IN− in a given IN-PC pair. The occurrence of IN-PC inhibition and CF-IN+ in a given pair was independent of one another (p = 0.13), whereas the association between IN-PC inhibition and CF-IN− was significant (p = 0.01, two-tailed Fisher’s exact tests). (E) Diagram of the resulting microcircuit motif.

To identify feedforward motifs (Figure 4A), we compared the effects on PC spiking exerted by INs with significant CF-IN excitation only and CF-IN inhibition only (Figure 4B). INs that were inhibited by the CF produced stronger IN-PC inhibition than those that were excited by the CF (normalized PC spike probability of 0.70 ± 0.13, n = 8, versus 0.90 ± 0.21, n = 13 after IN spike, p = 0.018) (Figure 4C). Moreover, the probability of observing significant IN-PC inhibition did not depend on the presence of CF-IN excitation in a given pair (3/13 pairs with CF-IN excitation exhibited significant IN-PC inhibition, p = 0.13), whereas the probability of observing IN-PC inhibition was increased in CF-IN inhibition pairs (7/8 pairs, p = 0.01, Fisher’s exact test) (Figure 4D). Contrary to predictions from *in vitro* studies, we thus revealed that INs activated by glutamate spillover from local CF activity show little direct effect on PC spike output, arguing against a crucial role of CF-PC feedforward inhibition via INs in controlling PC spiking. Instead, we demonstrate that the INs indirectly inhibited by CF input have the strongest impact on PC spike output, revealing a preference for a local CF-PC feedforward disinhibition motif via INs (Figure 4E).

### Millisecond Recruitment of Precisely Timed Feedforward Inhibition by Sensory Stimulation

**Figure 5:**
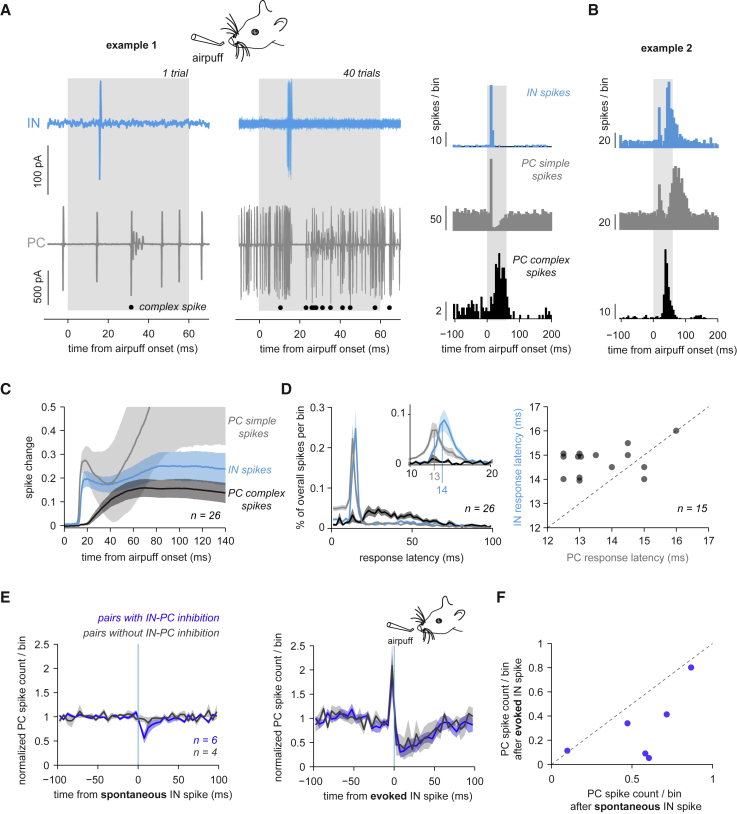
Millisecond Recruitment of IN-PC Inhibition by Sensory Stimulation (A) Example of a single trial of simultaneously recorded IN and PC responses to an airpuff directed at the ipsilateral whisker pad. Left: recording of IN (top) and PC (bottom) activity. Gray rectangle indicates airpuff duration. Black circles indicate PC complex spikes. Middle: same as left but showing 40 consecutive trials overlaid. Right: PSTHs (peri-stimulus time histograms) of IN spikes (top), PC simple spikes (middle), and PC complex spikes (bottom). Bin size = 5 ms. (B) Same as (A, right) but for another IN-PC pair. (C) Mean spike changes across all IN-PC pairs, i.e., mean number of delta spikes per stimulus, for IN spikes (blue), PC simple spikes (gray), and PC complex spikes (black). Shadings denote ±SEM. (D) Left: mean distributions of latencies from stimulus onset to first spike for IN spikes, PC simple spikes and complex spikes across all IN-PC pairs. Bin size = 2 ms. Inset: bin size = 0.5 ms. Shadings denote ±SEM. Right: PC response latencies plotted versus IN response latencies. Values are maxima of response latency distributions (bin size = 0.5 ms). (E) Left: IN-PC pairs were grouped into connected (blue, n = 6) and unconnected (gray, n = 4) pairs based on baseline-normalized PC spike counts/bin after spontaneous IN spikes. Right: for the same groups, mean baseline-normalized PC spike counts/bin aligned to IN spikes recruited by rapid granule-cell input, i.e., within 20 ms of stimulus onset. Trials with sensory-evoked complex spikes were removed. Shadings denote ±SEM. Bin size = 5 ms. (F) Amplitudes of spontaneous and sensory-evoked IN-PC inhibition plotted against each other for each connected pair. Amplitudes are measured as mean baseline-normalized PC spike counts/bin 5–10 ms post-IN spike. The dotted line represents the unity line.

To examine how the inhibitory circuitry is engaged during sensory processing, we delivered ipsilateral airpuffs to the whisker field or perioral region during simultaneous recordings from INs and PCs in crus II, an area known to receive tactile projections both via mossy-fiber-granule-cell inputs and via CFs (Brown and Bower, 2001). Sensory stimulation produced rapid activation of both INs and PCs (Figure 5), reflecting excitation by granule cells. This co-activation of INs and PCs was often rapidly followed by inhibition of PC simple spikes (Figure 5A) consistent with feedforward inhibition (Mittmann et al., 2005). PC complex spike responses occurred at longer and more variable latencies (Brown and Bower, 2001, Kitamura and Häusser, 2011). In some recordings, PC simple spikes showed additional delayed excitatory responses, as observed previously (Bosman et al., 2010). Surprisingly, we also found delayed excitatory responses in some INs, with a latency following simultaneously recorded PC complex spike responses (example 2 in Figure 5B: IN response latency peaks at 19 and 47 ms and PC complex spike response latency peak at 39 ms). This delayed sensory-evoked IN response was also obvious in the mean spike changes to the stimulus, i.e., the mean number of spikes that were added or omitted due to the stimulus per trial as a function of time (Figure 5C): after an early IN spike change with timing similar to that of rapid PC responses, the average IN spike change showed a delayed, slow increase following the average PC complex spike change.

We next investigated the exact temporal relationship of the initial IN and PC responses to tactile stimulation by calculating the distributions of latencies from stimulus onset to first spike (Figure 5D). On average, fast PC simple spike responses preceded fast IN responses by 1 ms, with the peaks of the response latency distributions positioned at 13 and 14 ms, respectively (n = 26). Accordingly, in pairs with distinct peaks in the response latency distributions for both IN and PC (see STAR Methods), we found that in most (n = 12/15), the IN response peaked ∼1 ms after the PC response (Figure 5D, right) (13.7 ± 1.1 versus 14.8 ± 0.6 ms for PCs and INs, respectively; p = 0.003, n = 15). INs and PCs are therefore recruited rapidly and near-synchronously by sensory-evoked granule-cell input.

What is the relationship between spontaneous IN-PC inhibition and sensory-evoked IN-PC recruitment and inhibition? To address this, we grouped dual recordings into connected and unconnected IN-PC pairs based on the presence of significant PC inhibition after spontaneous IN spikes (Figure 5E, left) and calculated IN-PC cross-correlograms aligned to sensory-evoked IN spikes, removing trials with sensory-evoked PC complex spikes. For connected and unconnected IN-PC pairs, the sensory-evoked cross-correlograms were similar, exhibiting sharp PC activation preceding the IN spike and prolonged PC inhibition afterward (Figure 5E, middle). The peak close to 0 ms lag in both groups shows that both connected and unconnected IN-PC pairs were recruited near-simultaneously by sensory-evoked granule-cell input. The subsequent PC inhibition was of similar magnitude in both groups (mean normalized PC spike probability in a 5–10 ms window post-IN spike of 0.30 ± 0.29, n = 6 pairs with spontaneous IN-PC inhibition, versus 0.45 ± 0.44, n = 4 pairs without spontaneous IN-PC inhibition, p = 0.352). Sensory-evoked IN-PC inhibition was more potent than spontaneous IN-PC inhibition across pairs (mean normalized PC spike probability post-IN spike of 0.36 ± 0.34 versus 0.71 ± 0.29 for evoked versus spontaneous IN-PC effects, respectively; p = 0.012) (Figure 5F). Moreover, sensory-evoked inhibition was prolonged in time, with decreased PC spiking probability up to ∼40 ms after the IN spike, whereas spontaneous IN-PC inhibition lasted up to ∼10 ms. Thus, during sensory processing, IN-PC cross-correlograms are similar for both connected and unconnected IN-PC pairs, exhibiting synchronization from granule-cell input followed by PC inhibition. These data suggest that sensory stimulation synchronizes large numbers of INs and PCs, with increased IN activation resulting in more potent PC inhibition.

### The Contribution of Single INs to Sensory-Evoked Feedforward Inhibition

**Figure 6:**
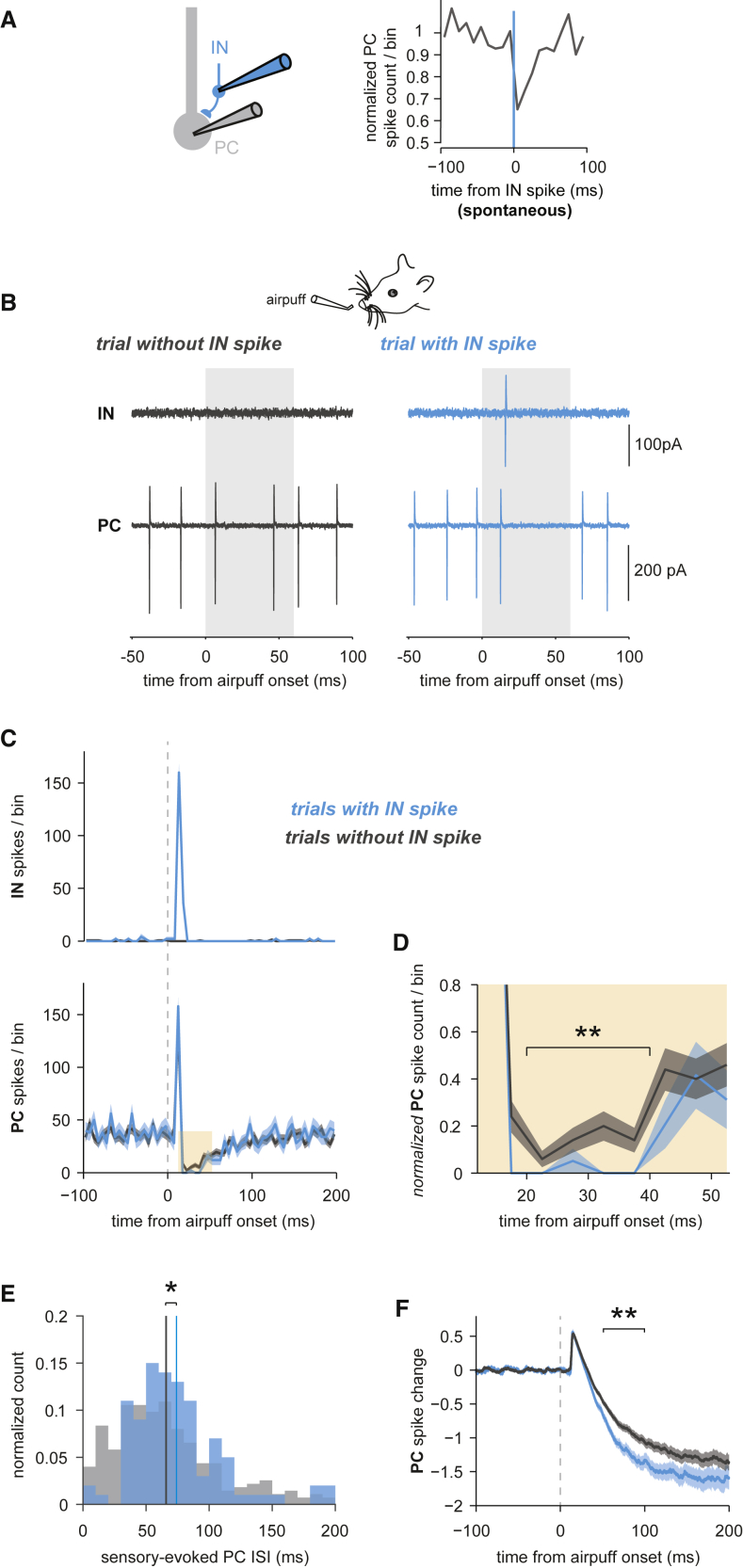
The Contribution of Single INs to Sensory-Evoked Feedforward Inhibition (A) Left: recording configuration. Right: Mean PC spike counts aligned to spontaneous IN spikes indicating IN-PC inhibition. (B) In the same IN-PC pair as shown in (A), sensory stimulation trials were categorized into trials without (gray) and trials with (blue) sensory-evoked rapid IN spikes. Single trials are shown. (C) PSTHs of IN spikes (top panel) and of PC simple spikes (bottom panel) for the two categories defined in (B). Trials with sensory-evoked complex spikes were removed. Shadings denote ±SEM. (D) Zoom-in on the bottom plot in (C) as indicated by the orange shading. Brackets indicate the temporal window in which the significance of difference in PC inhibition with and without sensory responses in the simultaneously recorded IN was assessed (20–40 ms postairpuff onset, p = 0.0037, Wilcoxon rank-sum test). (E) Distributions of PC ISIs around the mean time point of sensory-evoked IN responses for the same categories as in (B) and (C). Bin size = 10 ms. Vertical lines indicate distribution means (p = 0.015, Wilcoxon rank-sum test). (F) PC spike changes corresponding to same categories as in (B)–(D). Brackets indicate the time window in which the significance of difference between PC spike changes with and without sensory-evoked IN spikes was assessed (50–100 ms postairpuff onset, p = 0.0021, Wilcoxon rank-sum test). Shadings denote ±SEM.

To estimate the impact of single INs on PC inhibition recruited by sensory stimulation, we sorted sensory stimulation trials into trials with and without fast, granule-cell-evoked IN responses and compared PC inhibition in these two conditions (Figures 6A–6C). Sensory-evoked PC inhibition was significantly larger in trials with evoked IN spikes, reflecting the additional inhibition contributed by the recorded IN (mean normalized PC spiking probability in a 20–40 ms window after airpuff of 0.13 ± 0.41, n = 275 trials without IN spike, versus 0.01 ± 0.13, n = 100 trials with IN spike, p = 0.0037) (Figure 6D). Notably, this effect was only observed in pairs that were connected based on spontaneous IN-PC cross-correlograms (2/6), but not in unconnected pairs (0/4). We also examined the effect of single IN spikes on the ISIs of connected postsynaptic PCs. Trials in which the recorded IN responded to sensory stimulation had a significantly longer ISI compared with trials in which the recorded IN did not spike (mean PC ISIs 74 ± 37 versus 66 ± 42 ms, p = 0.015) (Figure 6E). This indicates that a spike in a single IN can prolong the spiking of the PC during a sensory-evoked response, paralleling the observations of single IN spikes on ISIs during spontaneous spiking (Figures S4B–S4D). Fast excitatory PC responses preceding PC inhibition were identical in trials with and trials without IN recruitment (Figure 6F), indicating that granule-cell-mediated IN and PC responses were independent of each other trial by trial. Thus, although several synchronously active INs are involved in generating the prominent PC inhibition upon sensory stimulation, the recruitment of a presynaptic IN on a given trial is detectable in the inhibition of the PC, highlighting the impact of single INs on PC output.

### The Recruitment of INs during Sensory Processing Is Pathway Specific and Spatially Organized by IN Depth in the Molecular Layer

**Figure 7:**
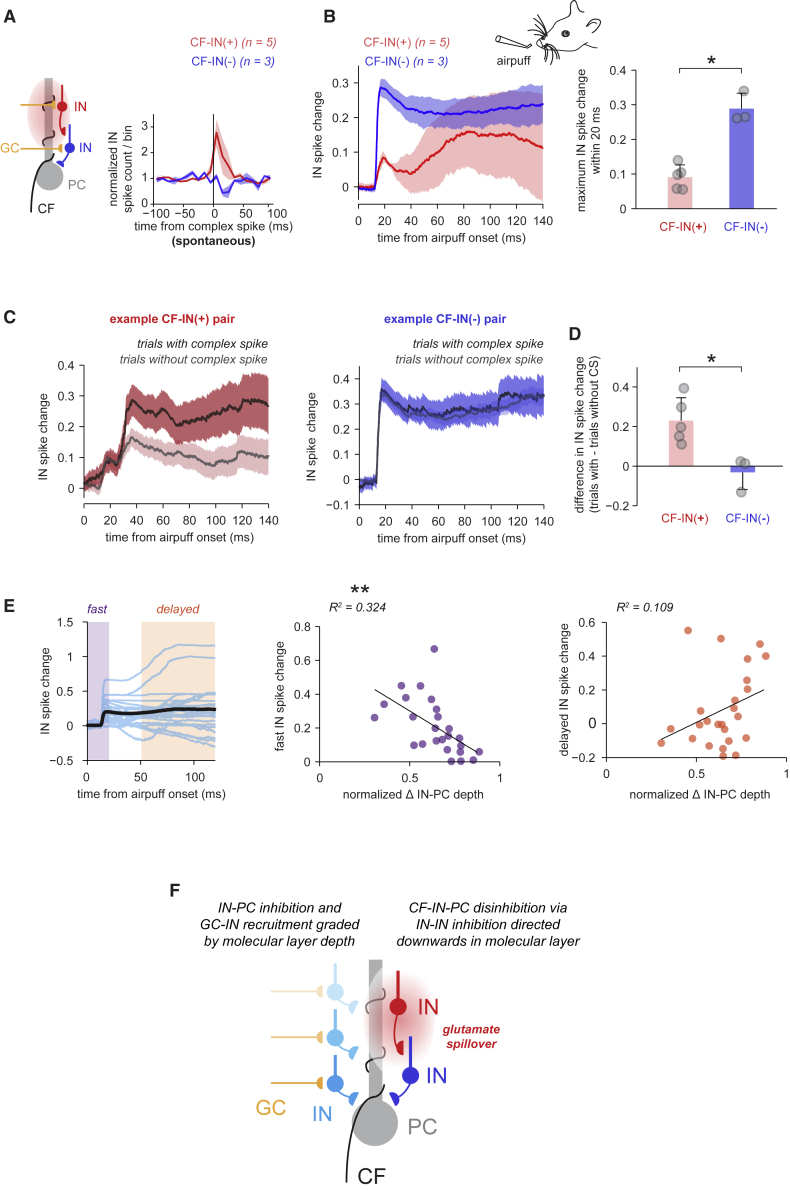
The Recruitment of INs during Sensory Processing Is Pathway-Specific and Spatially Organized by IN Depth in the Molecular Layer (A) Left: diagram of the relevant microcircuit. Red: INs with excitation after spontaneous CF input (CF-IN+, n = 5), blue: INs with inhibition (CF-IN−, n = 3). Right: normalized mean IN spike counts/bin aligned to spontaneous PC complex spikes are used for IN grouping. Shadings denote ±SEM. (B) Left: mean IN spike changes after an airpuff for the same IN groups as in (A). Right: comparison of maximum IN spike change within 20 ms after airpuff onset for the CF-IN+ and CF-IN− group (mean + SD, p = 0.036, Wilcoxon rank-sum test). (C) Left: example of airpuff-evoked mean spike changes in a single IN (CF-IN+ type) for trials with simultaneously recorded sensory-evoked PC complex spikes (black/dark red) and trials without (gray/light red). Note the larger secondary IN response in trials with local PC complex spikes. Right: same as left, but for another recording with IN inhibition after spontaneous CF input (CF-IN− type). Note the absence of delayed secondary sensory responses. Shadings denote ±SEM. (D) Differences of IN spike changes 0–140 ms from airpuff onset between trials with and trials without local PC complex spikes for same IN groups as in (A) and (B) (mean + SD, p = 0.036, Wilcoxon rank-sum test). (E) Left: individual (blue) and mean (black) IN sensory-evoked spike changes (n = 26 INs). IN spike changes were separated into a fast component (0–20 ms from airpuff onset, purple) and a delayed component (50–120 ms from airpuff onset, orange). Middle: amplitude of fast IN spike change plotted versus IN depth in the molecular layer. Black line: linear regression line, p = 0.003. Right: amplitude of delayed IN spike change (difference between delayed and fast amplitude) plotted versus IN molecular-layer position. Black line: regression line, p = 0.107. (F) Summary diagrams of IN microcircuit principles organized by IN depth in the molecular layer.

Given that in some pairs delayed IN sensory responses occurred with timecourses similar to those of sensory-evoked CF input (Figures 5B and 5C), we investigated the relationship between CF and IN activity during sensory processing. We grouped INs according to their spike modulation upon spontaneous CF input into INs responding with spike probability increases and decreases (Figure 7A). The average sensory responses in these IN groups exhibited clear differences (Figure 7B): INs that were inhibited by spontaneous CF input responded to sensory stimulation with a large, fast excitatory component, whereas INs excited by CF input showed weaker fast activation (maximum IN spike change within 20 ms after airpuff onset of 0.29 ± 0.05, n = 3, versus 0.09 ± 0.04, n = 5, p = 0.036). The CF-IN excitation group showed an additional slow excitatory response component that was absent from the CF-IN inhibition group.

To unmask the contribution of CF input to sensory responses in these IN groups, we quantified sensory responses of INs in trials with and without sensory-evoked local CF input, measured by the presence of PC complex spikes. Figure 7C (left) shows an example of this analysis in an IN with spontaneous CF-IN excitation: although the fast IN response was not affected, the slow response was bigger in trials with sensory-evoked CF input, suggesting that local CF input contributed to delayed excitatory sensory responses in INs. The second component was smaller but still present in trials without CF input to the local PC, which suggests that the IN may pool input from several CFs (Szapiro and Barbour, 2007) that did not all respond on a given trial. The second, slow response was absent from INs with spontaneous CF-IN inhibition, irrespective of local CF recruitment (example in Figure 7C, right), suggesting that even during sensory processing, when CF input is known to be more synchronized, INs with spontaneous CF-IN inhibition successfully avoid CF excitation. The difference in IN spike change between trials with and those without local CF input was positive for pairs with spontaneous CF-IN excitation, but not for pairs with CF-IN inhibition, and it differed significantly between these two groups (Figure 7D) (mean differences of 0.23 ± 0.12, n = 5, versus −0.03 ± 0.09, n = 3, for CF-IN increase and decrease groups, respectively; p = 0.036), highlighting the role of CF input in segregating IN activity during sensory processing. These results demonstrate that INs are recruited differentially by sensory-evoked granule-cell and CF input, depending on the sign of their response to spontaneous CF input.

Given that IN-PC inhibition, IN-IN inhibition, and the resulting CF-IN polysynaptic inhibition were all organized by IN depth in the molecular layer, we asked whether sensory-evoked excitatory recruitment also depends on somatic IN depth. We again categorized IN airpuff responses into a fast, granule-cell-mediated component and a slow, CF-mediated component (Figure 7E). We found that the amplitude of the fast component scaled inversely with the depth of the IN in the molecular layer; i.e., deeper INs responded more strongly (Figure 7E) (R^2^ = 0.32, p = 0.003). The delayed IN response component did not depend on IN depth in the molecular layer, matching the broad spatial profile of spontaneous CF-IN excitation (R^2^ = 0.109, p = 0.107). In summary, we show that IN somatic depth in the molecular layer is an organizing principle for microcircuit rules governing the recruitment and the impact of single INs on their targets (Figure 7F).

## Discussion

We made simultaneous recordings from INs and PCs *in vivo* to determine the functional impact of single INs on their targets and to identify the circuit rules governing the impact of individual connections. We show that individual spikes in single INs can inhibit their targets potently, with the efficacy of inhibition being stronger for proximal INs. Furthermore, an IN’s response to local CF input dictates its effect on PC spiking: INs excited by CF input have little direct influence on PC simple spike output but cause delayed inhibition of a separate IN population that controls PC output. This segregation of IN activity is conserved during sensory processing, with INs inhibited by CF input being more strongly activated by granule-cell input and INs excited by CFs showing additional delayed responses. These results reveal the circuit building blocks for sculpting PC responses to sensory stimulation. The differential engagement of INs over a range of timescales, via different pathways, allows PC activity to be both precisely timed (Brown and Raman, 2018, Heck et al., 2007) and modulated over longer timescales (Chabrol et al., 2019, Gao et al., 2018, Sarnaik and Raman, 2018).

### The Functional Impact of Single INs on Spike Output *In Vivo*

Our findings provide direct evidence that single spikes in single identified INs in the mammalian brain can produce significant inhibition of principal cell spiking *in vivo*. Although there are previous reports of inhibitory interactions inferred from spike trains of extracellularly recorded INs and principal neurons in several brain areas (Barthó et al., 2004, Blot et al., 2016, Diba et al., 2014), these studies lacked the ability to directly stimulate the neuron presumed to cause the inhibition and are thereby restricted to reporting correlations. By triggering spikes in single INs, we directly assessed the postsynaptic impact of a single spike in a single IN and demonstrated that this causes brief but potent inhibition of neighboring PC spiking. The strength of single IN inhibition we observe is surprising given that modeling studies have predicted substantially weakened impact of individual synaptic inputs *in vivo* (Bernander et al., 1991, Rapp et al., 1992) due to high levels of background synaptic input in the intact brain (Destexhe and Paré, 1999, Powell et al., 2015, Waters and Helmchen, 2006).

PC inhibition from triggered IN spikes was comparable to PC inhibition aligned to spontaneous IN spikes at the same synaptic connection, demonstrating that cross-correlograms of spontaneous IN-PC spikes can be used to read out functional inhibitory connectivity between single IN-PC pairs. This also suggests that most INs do not fire in precise synchrony under spontaneous conditions, consistent with the low incidence of millisecond-synchronous spiking in our dual IN recordings. INs are spontaneously active, driven by intrinsic properties in the absence of excitatory inputs (Häusser and Clark, 1997, Raman and Bean, 1997), which likely contributes to this finding. Spontaneous synchrony may be further disrupted by GABAergic connectivity among INs.

### A Gradient of Inhibitory Efficacy Depending on IN Location

We systematically sampled the impact of INs located throughout the molecular layer and show that *in vivo*, the most proximal INs produce the most powerful inhibition of PC output. This result is in general accordance with the classical division of INs into stellate cells higher up in the molecular layer and basket cells proximal to the PC layer (Palay and Chan Palay, 1974), and with physiological studies showing that only the more proximal basket cells exert strong control of the PC spike rate (Bao et al., 2010, Blot and Barbour, 2014, Brown et al., 2019). However, rather than two distinct classes of IN-PC effects, we found a gradient of inhibitory efficacy across the entire depth of the molecular layer. This continuum in the functional impact across the IN population may have an anatomical basis in the varying numbers and locations of IN axonal collaterals (Sultan and Bower, 1998).

The sharp distance dependence of IN-PC interactions in the transverse plane—with no connections detectable at a 40 μm distance—matches morphological constraints on IN axon connectivity (Palay and Chan-Palay, 1974), supporting that INs mainly deliver local inhibition within a given sagittal plane. In contrast, in the sagittal plane, IN axons can extend for hundreds of micrometers, allowing them to mediate lateral inhibition (Cohen and Yarom, 2000, Dizon and Khodakhah, 2011, Eccles et al., 1967, Sultan and Bower, 1998). Our data showing no decrease of functional IN-PC connectivity within the first 100 μm in the sagittal axis are consistent with this axonal morphology.

### Sensory Stimulation Rapidly Recruits Feedforward Inhibition from INs onto PCs

We demonstrate that INs are rapidly recruited by sensory-driven granule-cell input, exhibiting millisecond-precise synchrony with the activation of PCs. This highlights the role of feedforward inhibition in terminating excitation of PCs after granule-cell input (Mittmann et al., 2005), narrowing the time window for integration of excitatory inputs. Importantly, even single INs engaged by the sensory stimulation can contribute significantly to the inhibition of postsynaptic PCs, as measured by the overall impact on PC spiking and on PC ISIs following the sensory stimulus. The finding that tactile granule-cell recruitment more strongly activates INs deep in the molecular layer, which are the INs inhibiting PC spiking most efficiently, supports this notion (Chu et al., 2012). In concert with ephaptic coupling between PCs (Han et al., 2018), this rapid engagement of IN inhibition may help ensure well-timed spikes in response to sensory stimulation (Brown and Raman, 2018) and the millisecond synchrony of PC spiking during a learned reaching task, precisely locked to movement (Heck et al., 2007).

Sensory-evoked responses in PCs were often ∼1 ms quicker than IN responses to the same sensory stimulus, despite INs being smaller and having higher input resistances. The faster response of PCs may result from a combination of factors: sensory information could be mediated mainly by granule-cell-ascending axons instead of parallel fibers (Gundappa-Sulur et al., 1999). Alternatively, PCs receive input from more synchronously active granule-cell inputs than INs and, moreover, have shorter ISIs during spontaneous firing and thus are brought to spike threshold more quickly. Prolonged PC inhibition during sensory processing may stem from effects of INs activated by delayed CF input to neighboring PCs, synergistically prolonging PC inhibition (even if most of those INs do not have measurable effects on PC spiking by themselves). Alternatively, long PC spike decreases may result from cell-intrinsic plasticity mechanisms (Johansson et al., 2015), complicating the interpretation of PC sensory responses.

During sensory processing, we find that functionally connected and unconnected IN-PC pairs exhibit similar cross-correlograms indicative of feedforward inhibition. Sensory stimulation thus activates many INs and PCs in synchrony whose firing is uncorrelated during spontaneous activity. This finding also suggests that the anesthetized state, with low spontaneous granule-cell activity (Wilms and Häusser, 2015), may represent a favorable setting for inferring inhibitory connectivity from spontaneous IN-PC cross-correlograms, whereas during sensory processing or in the awake state, increased network synchrony may render cross-correlograms between connected and unconnected IN-PC pairs indistinguishable without the ability to reliably categorize spikes as spontaneous or sensory-evoked. Nevertheless, given that isoflurane modulates GABA receptors allosterically (Garcia et al., 2010), and in light of the richness of neuromodulatory projections to cerebellar cortex (Libster and Yarom, 2013), it will be important to investigate the state dependence of IN-PC inhibition in the awake animal.

### CF-IN Interactions Mediated by Glutamate Spillover *In Vivo*

Although previous *in vivo* studies reported excitatory IN responses to electrical inferior olive stimulation (Eccles et al., 1966, Jirenhed et al., 2013, Jörntell and Ekerot, 2003), optogenetic CF activation (Rowan et al., 2018), and similar time courses of CF and IN activity during cerebellar-dependent behavior (Badura et al., 2013, ten Brinke et al., 2015), simultaneous measurements of IN spiking and physiological CF activity have been lacking. We demonstrate that CF input can activate INs via an unconventional glutamate spillover pathway (Coddington et al., 2013, Mathews et al., 2012, Szapiro and Barbour, 2007), which is surprising given various mechanisms working against spillover transmission *in vivo* (Asztely et al., 1997, Auger and Attwell, 2000, Dzubay and Jahr, 1999). Not all INs were activated by CF input: rather, INs located deep in the molecular layer tend to be inhibited, most likely via disynaptic inhibition from more distal INs. This motif is consistent with inhibitory connections within the IN network being directed downward in the molecular layer (Rieubland et al., 2014), a spatial organization we also observed in our dual IN recordings.

A similar microcircuit structure has been described in other brain areas: in neocortex, foot shocks used during auditory fear conditioning recruit basal forebrain afferents activating layer 1 INs, which inhibit PV^+^ INs below them, resulting in principal cell disinhibition (Letzkus et al., 2011). A comparable circuit logic has been found in the basolateral amygdala (Wolff et al., 2014). In cerebellar associative learning, the unconditioned stimuli (or error signals) are thought to activate the CF pathway (Ohmae and Medina, 2015, ten Brinke et al., 2015, Yeo and Hesslow, 1998). Thus, inhibition of deep INs by unconditioned stimuli appears to be a common motif across brain areas.

### A Preference for CF Feedforward Disinhibition of PCs via INs

The effect of CF-IN transmission on PC activity is usually considered in a feedforward inhibitory framework in which activated INs inhibit the local PC, prolonging postcomplex spike pauses (Marshall and Lang, 2009, Mathews et al., 2012, Wulff et al., 2009). In contrast, our data reveal a microcircuit in which INs inhibited upon CF input inhibit spiking of the local PC, thereby forming a motif of local PC disinhibition upon CF input. Via disinhibition, the IN population may have a role in setting a time window for the reset of PC simple spiking after a complex spike, modulating PC simple spike facilitation observed *in vivo* (De Zeeuw et al., 2011, Marshall and Lang, 2009). Somatic disinhibition may also control variability of PC spiking after CF input by affecting the dynamic, complex interactions between somatic and dendritic PC currents (Jaeger et al., 1997).

Activation of vertically distant INs may provide dendritic inhibition to PCs that we could not assess by recording somatic PC spikes. In support of this, inhibition has been shown to regulate variability of CF-triggered dendritic calcium levels (Kitamura and Häusser, 2011), and IN activity can regulate CF-evoked dendritic calcium levels and thereby granule-cell PC synaptic plasticity (Callaway et al., 1995, Gaffield et al., 2018, Rowan et al., 2018).

### Implications for Plasticity

Albus (1971) hypothesized that CF input drives plasticity not only at the granule-cell-PC synapse but also at the granule-cell-IN synapse. Moreover, he postulated that different INs should exhibit opposite plasticity rules with CF input (Albus, 1971), increasing the network’s computational capacity. By demonstrating that different INs are activated and inhibited by CF input *in vivo*, our data suggest opposite changes in the IN responses to CF input as a possible candidate mechanism for the implementation of such learning rules. CF input can shape IN and PC receptive fields (Jörntell and Ekerot, 2002), and IN membrane potentials following granule-cell input can affect synaptic plasticity (Rancillac and Crépel, 2004), supporting that CF-triggered plasticity of granule-cell-IN synapses can follow diverse rules (Albus, 1971). Our results describing sensory-evoked IN responses are in line with this hypothesis: INs exhibited not only fast, granule-cell-mediated activation but also delayed activation correlated with CF input. Interestingly, granule-cell-mediated IN responses were larger in INs without delayed CF-associated IN responses, suggesting that sensory-evoked CF-IN excitation may affect granule-cell-IN synaptic strength. During sensory processing, the inhibition of INs by CF input in a subset of powerful INs might be crucial to preserve balanced activation of these INs and their target PCs by the same granule-cell inputs, serving a homeostatic function and maintaining granule-cell feedforward inhibition over long timescales.

In summary, our results reveal the impact of single cerebellar INs on their targets under *in vivo* conditions and dissect the circuit logic underlying the recruitment of INs by different excitatory inputs. These results provide critical constraints for any model of cerebellar function and should inspire future studies on the dynamics of this microcircuit during skilled behavior and learning.

## STAR★Methods

### Key Resources Table

**Table:** 

REAGENT or RESOURCE	SOURCE	IDENTIFIER
**Chemicals, Peptides, and Recombinant Proteins**		

Chlorprothixene Hydrochloride	Sigma	C1671-1G
Dexamethasone	Norbrook	Colvasone
Alexa Fluor 594 Hydrazide	Thermo Fisher Scientific	A10438
Sodium chloride	Sigma	S6191
Potassium chloride	Sigma	P9333
HEPES	Sigma	H3375
Calcium Chloride	VWR	190464K
Magnesium Chloride	Sigma	63069
Agarose	Sigma	A6138

**Experimental Models: Organisms/Strains**		

*Mouse*: PV-eGFP	Hannah Monyer, Meyer et al., 2002;	N/A

**Software and Algorithms**		

Axograph	Axograph Scientific	https://axograph.com/
FIJI	Schneider et al., 2012	https://fiji.sc/
MATLAB	MATHWORKS	https://www.mathworks.com/
ScanImage	Pologruto et al., 2003	https://vidriotechnologies.com/scanimage/
Graphpad	Graphpad Software	https://www.graphpad.com/quickcalcs/

**Other**		

Laser	Spectra-Physics	Mai Tai HP
Objective	Nikon	CFI75 LWD 16X W
Amplifier	Molecular Devices	Multiclamp 700B
Micropipette puller	Narishige	PC-10
Capillary glass	World Precision Instruments	1B150F-4

### Lead Contact and Materials Availability

Further information and requests for resources and reagents should be directed to and will be fulfilled by the Lead Contact, Michael Hausser (m.hausser@ucl.ac.uk). This study did not generate new unique reagents.

### Experimental Model and Subject Details

Animal procedures were performed under license from the UK Home Office in accordance with the Animal (Scientific Procedures) Act 1986. 52 adult (P30-60) male and female (ca 50% each) transgenic mice expressing EGFP in parvalbumin-positive neurons (Meyer et al., 2002) were used to identify and target molecular layer interneurons (INs) and Purkinje cells (PCs). Mice were group-housed and kept on a regular light-dark cycle. Experiments were performed during the light cycle of the mice.

### Method Details

#### Surgical Preparation

Mice were anaesthetized with isoflurane (2% during surgery, 0.75% during recordings) and injected intraperitoneally with chlorprothixene (1 mg/kg) and dexamethasone (1 mg/kg). Body temperature was monitored using a rectal probe and maintained at 37.2 ± 0.2°C using a feedback-controlled heating blanket. Craniotomies (1 mm^2^) were made over vermis lobule 5 (medial) or Crus II. The dura was left intact. If necessary, the brain was covered using 1.5% agar in HEPES buffered artificial cerebrospinal fluid (ACSF: 150 mM NaCl, 2.5 mM KCl, 10 mM HEPES, 2 mM CaCl_2_ and 1 mM MgCl_2_, pH 7.3) to reduce brain movement.

#### *In vivo* dual patch-clamp recordings

*In vivo* targeted loose patch-clamp recordings (Kitamura et al., 2008, Margrie et al., 2003) were performed using a custom two-photon microscope (MOM, Sutter) with a Ti:Sapphire laser (Mai Tai; Spectra-Physics) and a 16X, 0.8 numerical aperture water-immersion objective (Nikon). Images were acquired using ScanImage (Pologruto et al., 2003) in conjunction with MATLAB (Mathworks). An excitation wavelength of 865 nm was used to visualize EGFP-positive neurons. The pipette solution contained ACSF and 20 μM Alexa Fluor 594 (Thermo Fisher Scientific). Recordings were made using a Multiclamp 700B amplifier (Molecular Devices). Data were filtered at 6-10 kHz and acquired at 20 kHz in voltage clamp using an ITC-18 board (Instrutech) in conjunction with Axograph software (Axograph Scientific). Patch pipettes were pulled using a vertical puller (Narishige) from thick walled borosilicate glass and had a resistance of 5 or 7 MΩ for PC and IN recordings, respectively. Spontaneous spiking activity was recorded for 5-25 minutes (n = 56 IN-PC pairs and 28 IN-IN pairs). To trigger single spikes in INs (n = 30 IN-PC pairs), a 200 mV, 0.2 ms voltage pulse was delivered through the IN recording pipette at a rate of 1 Hz. Sensory stimulation was performed by delivering a 60 ms airpuff (60 PSI) to the ipsilateral perioral region and/or whisker pad (Picospritzer III, Parker) with an inter-stimulus interval of 1- 2 s controlled with Axograph (n = 26 IN-PC pairs). The position of the airpuff was adjusted until sensory responses in the IN and/or PC were observed. After each recording, a z stack of IN and PC pipette position (256 x 256 pixels) was recorded at 3 μm z resolution.

### Quantification and statistical analysis

#### Spike train cross-correlation analysis

All analysis was performed using custom-written software in MATLAB. Spikes were detected with a manually set amplitude threshold. To distinguish simple and complex spikes, the variance 3 ms following the spike was plotted and the resulting two populations were separated with a manual threshold. Cross-correlograms between two spike trains were calculated by using spike times of one trace as the triggers and calculating the average spike count per bin of the target spike train 100 ms before and after the trigger. For IN-PC simple spike cross-correlograms, IN spikes following a complex spike within 25 ms were excluded as trigger spikes. Cross-correlograms were tested for significance as follows: trigger spike times were shuffled by reordering 5 s periods of the recording randomly, and 500 shuffled cross-correlograms (10 ms bin size) were calculated based on the shuffled trigger times. To avoid artifacts from non-stationarities, the original and shuffled cross-correlograms were normalized to their respective mean baseline rate (for PCs: –100 to –80 ms from IN spike, for INs: –100 to 0 ms from complex spike), and the standard deviations of the shuffled cross-correlograms were normalized to the original’s baseline rate, before a standard score (z-score) was calculated for each bin. Z-scores of > 3 or < –3 were considered significant. This method likely underestimates the number of functional monosynaptic connections *in vivo* as it does not report weak connections or electrotonically remote synapses on PC distal dendrites. To detect IN-PC inhibition, the 0-10 ms bin was tested for significance. For CF-IN excitation, effects were considered significant if either of the 0-10 or 10-20 ms bin exceeded the threshold. For CF-IN inhibition, effects were considered significant if either of the 10-20 or 20-30 ms bin exceeded the threshold. For IN-IN cross-correlograms, a 5 ms bin size was used. Co-activation was defined as z-score > 3 with ± 10 ms lag, inhibition was defined as z-score < −3 with ± 10 ms lag, and synchrony indicative of gap-junction coupling was tested by computing cross-correlograms with 0.5 ms bin size and looking for significant peaks at ± 1 ms lag.

#### Alternative quantifications of IN-PC inhibition

We also measured the amplitude of PC spike inhibition after spontaneous IN spikes in terms of net spike change, i.e., the average number of spikes added or omitted in response to a given event (see Figure S2). For IN-PC inhibition, we calculated the cumulative sum of all PC simple spikes from −100 to +100 ms from the IN spike times of all trials. From that, we subtracted a linear fit to the baseline period of −100 to −80 ms from the IN spike time, and divided the result by the number of trials to yield the net spike change. We found this quantity to depend on the PC baseline spike rate, unlike the baseline-normalized PC spike count per bin after an IN spike (see Figures S2F–S2H), and thus preferred to quantify inhibition as the baseline-normalized spike count per bin for the rest of the analyses. Excitatory spike changes in response to sensory stimuli, i.e., the number of added spikes after an airpuff, on the other hand, did not depend on baseline firing rates (data not shown), so we frequently used this measure to quantify sensory responses in detail.

We furthermore quantified electrically triggered IN-PC inhibition as an effect of the IN spike on the PC inter-spike interval (ISI) that the triggered IN spike fell into. To do so, we calculated the distribution of PC ISIs surrounding the real, triggered IN spikes and 1000 shuffled distributions of PC ISIs around randomly positioned hypothetical IN spike times, matching the number of random IN spike times to the number of triggered IN spikes in each shuffle. Then we compared the median of the real PC ISIs to a distribution of medians from the shuffles (Figure S4). This approach corroborated our IN-PC inhibition classification based on z-scored PC spike counts per bin, as z-scores calculated from effects on PC ISIs and z-scores calculated from PC spike counts per bin as described above were highly correlated. Accordingly, we found on average a significant increase of the PC ISI in the group of pairs with significantly reduced PC spike counts in the 0-10 ms bin after the IN spike, but not in the other group without significant PC spike count decreases (Figure S4).

#### Analysis of sensory-evoked spiking activity

To analyze sensory-evoked spike latencies, the distribution of latency to first spike after stimulus onset was calculated for each recording. To compare rapid IN and PC response timings per dual recording, only recordings in which the latency distributions for both IN spikes and PC simple spikes exhibited clear peaks (i.e., maximum values at least 4 standard deviations above mean latency) were included (n = 15/26 pairs). To analyze the amplitude of sensory-evoked IN spike changes, the cumulative sum of all spike times was calculated, a linear fit was fitted to the baseline (–200 to 0 ms from stimulus), extrapolated over the entire trace (+300 ms from stimulus), and subtracted (Mittmann and Häusser, 2007). Data are reported as mean ± standard error of the mean. When sorting sensory-evoked trials based on complex spike presence, all complex spikes occurring within 100 ms of stimulus onset were considered sensory-evoked. To isolate rapid, granule cell-mediated IN spike changes, a time window of 0-20 ms from stimulus onset was used. To measure the amplitude of delayed IN responses, a 50-120 ms time window post stimulus onset was used. The amplitude of sensory responses in terms of spike change was defined as the maximum of the mean spike change in a given time window. To obtain the net delayed IN spike change amplitude, the fast spike change amplitude was subtracted from the maximum of the mean spike change in the delayed response window. To compare spontaneous and sensory-evoked IN-PC inhibition, only pairs with sensory-evoked PC inhibition, a minimum of 20 trials with sensory-evoked IN spikes, and enough spontaneous IN-PC spikes for cross-correlation analysis (minimum of 200 spontaneous IN spikes) were used (n = 10). Accordingly, to relate spontaneous CF-IN effects to IN sensory responses, only pairs with recordings of sensory-evoked spikes and enough spontaneous spikes for cross-correlation analysis (minimum of 200 IN spikes within ± 100 ms of PC complex spikes) were used.

#### Two-photon stacks of pipette positions

Two-photon images were analyzed using Fiji (http://fiji.sc/) (Schneider et al., 2012). The two channels containing measurements of green and red fluorescence were assigned green and red lookup tables, respectively. The channels were overlaid, and the angle of the parasagittal plane in the images was measured by drawing a line along the orientation of PC dendrites seen as long stripes in the field of view. Images were rotated by the measured angle to align PC dendrites vertically (at 90 degrees). The identity of the neurons recorded from was clear from the pipette tip positions touching the neurons’ somata. Measurements of the positions of the somata centers, the PC layer and the dura mater were made in Fiji by placing cursors in the respective positions and planes. Data were then exported to MATLAB to calculate Euclidean intersomatic distances in the transverse and sagittal planes, and by molecular layer depth.

#### Statistical tests

For paired data, the Wilcoxon signed-rank test was used to assess significance. For unpaired data, the Wilcoxon rank sum test was used. For comparisons of mean values across more than two groups, the Kruskal-Wallis test plus multiple comparisons test was used. p < 0.05 was considered significant. For analysis of higher-order connectivity, Fisher’s exact test was calculated using the QuickCalc tool on https://www.graphpad.com/, the associated p values are two-tailed p values calculated using the method of summing small p values. In all Figures, ^∗^ p < 0.05, ^∗∗^ p < 0.01.

### Data and Code Availability

Analysis-specific code and datasets are available by request to the Lead Contact: m.hausser@ucl.ac.uk.

## Supplemental Information

Document S1. Figures S1–S9

Document S2. Article plus Supplemental Information
