# Supplementary material for: Microcircuit Rules Governing Impact of Single Interneurons on Purkinje Cell Output In Vivo
Source: Cell Rep. 2020 Mar 3;30(9):3020–3035.e3. doi: 10.1016/j.celrep.2020.02.009 (PMC7059114; doi:10.1016/j.celrep.2020.02.009)
Supplement: Document S1. Figures S1–S9 [file mmc1.pdf]

Cell Reports, Volume *30*

Supplemental Information

Microcircuit Rules Governing Impact of Single Interneurons on Purkinje Cell Output *In Vivo*

Charlotte Arlt and Michael Häusser

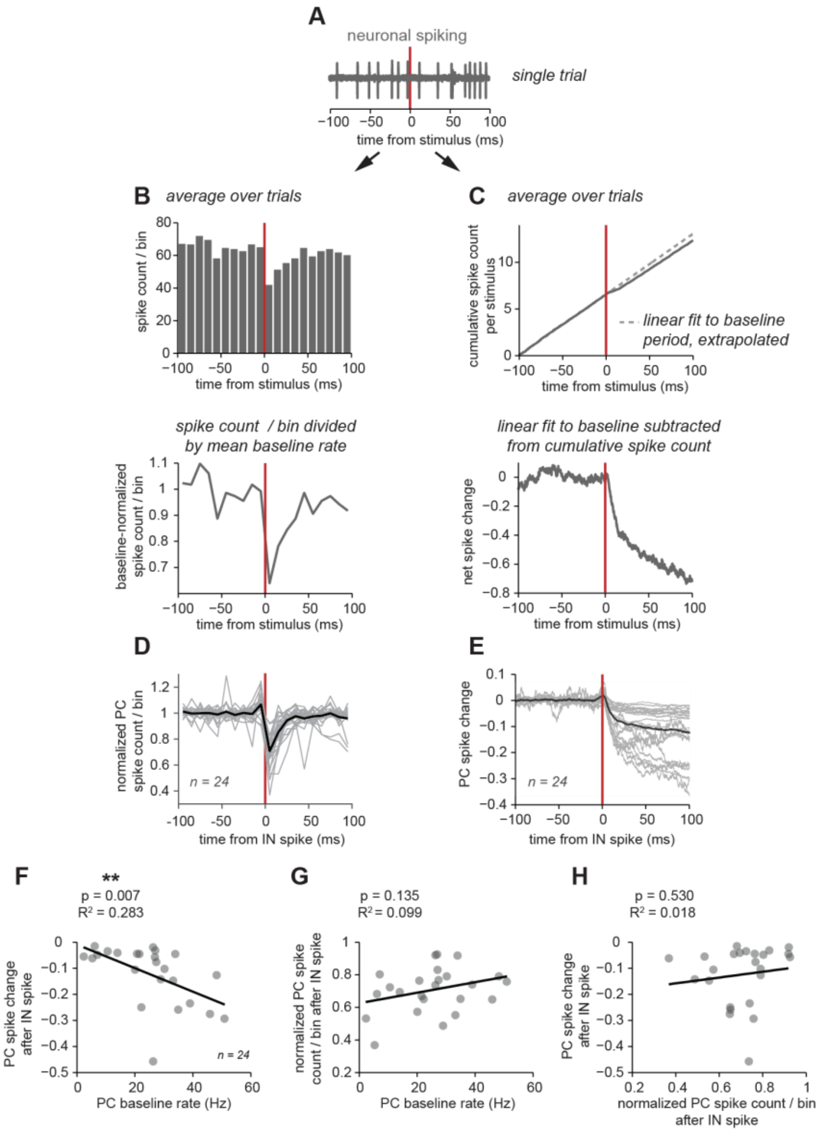

Figure S1. Quantifying IN-PC inhibition as changes in binned spike probability versus net spike change. Related to Figure 1

(A) Spikes of a given neuron (here: PC spikes) are aligned to the timing of a stimulus, e.g. a spike in a neighboring neuron (here: IN spikes) or a sensory stimulus, indicated by the red line. We compared two different metrics to quantify the neuron's response: (B) Average spike counts per bin (bin size = 10 ms). Below: as above, but normalized by the mean baseline spike rate. (C) Spikes from all trials are summed cumulatively, and the number is divided by the number of trials (grey solid trace). A linear fit to the baseline is extrapolated over the trial duration (grey dotted trace) and subtracted from the cumulative spike count to yield the net spike change (i.e. delta number of spikes). (D) Normalized PC spike count per bin for all IN-PC pairs with significant IN-PC inhibition. Grey: Individual pairs, black: Average. (E) Individual (grey) and average (black) PC spike changes after IN spikes, same pairs as in (D). (F) For the same pairs as in D and E, the net PC spike change after IN spikes is correlated with the PC baseline spike rate. Black: Linear regression line. (G) The baseline-normalized binned PC spike count after IN spikes does not linearly correlate with the PC baseline spike rate. (H) The measures of PC spike change and baseline-normalized binned spike count are not significantly correlated.

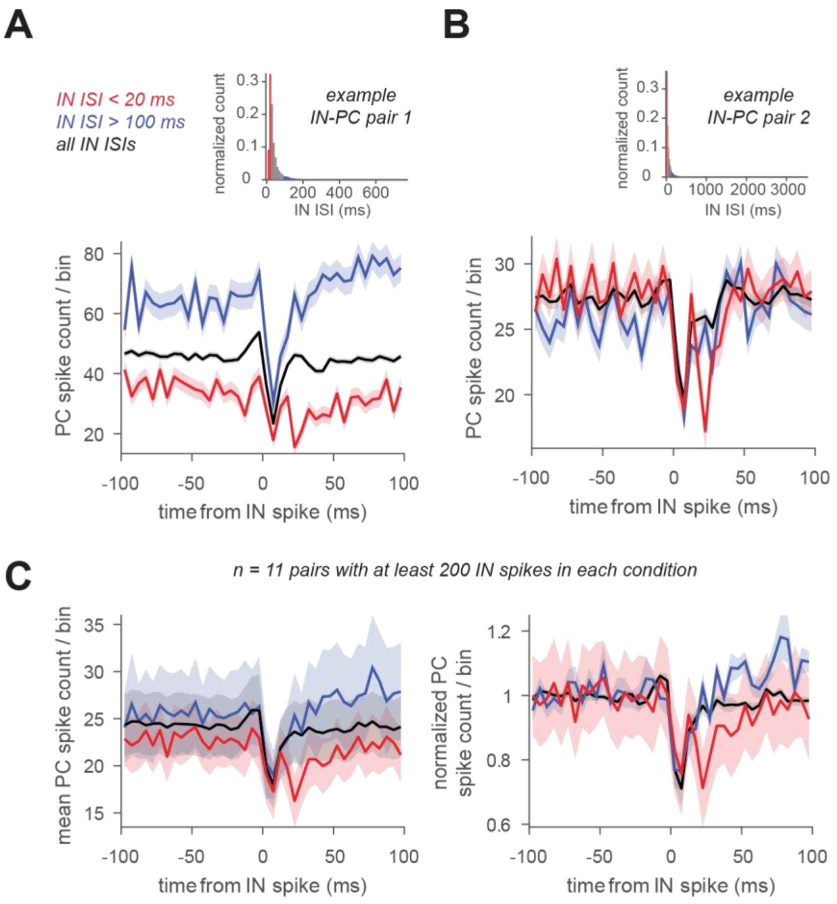

Figure S2. IN-PC inhibition for short and long IN ISIs. Related to Figure 1

(A) Example dual IN-PC recording. Top: IN spikes were categorized based on the subsequent ISI duration into short (< 20 ms, i.e. IN spike doublets, red color code) and long (> 100 ms, blue color code) ISIs. Bottom: Average PC spike count per bin aligned to either short, long, or all IN ISIs. Note the additional PC spike count decrease after IN spike doublets. (B) Same as A, but for a different IN-PC recording. (C) Left: Same as bottom row of (A ,B), but averaged across all IN-PC pairs with significant IN-PC inhibition and at least 200 IN spikes in each IN ISI category (n = 11 pairs). Right: Same as left, but after normalizing each condition in each IN-PC pair to the baseline PC spike count / bin.

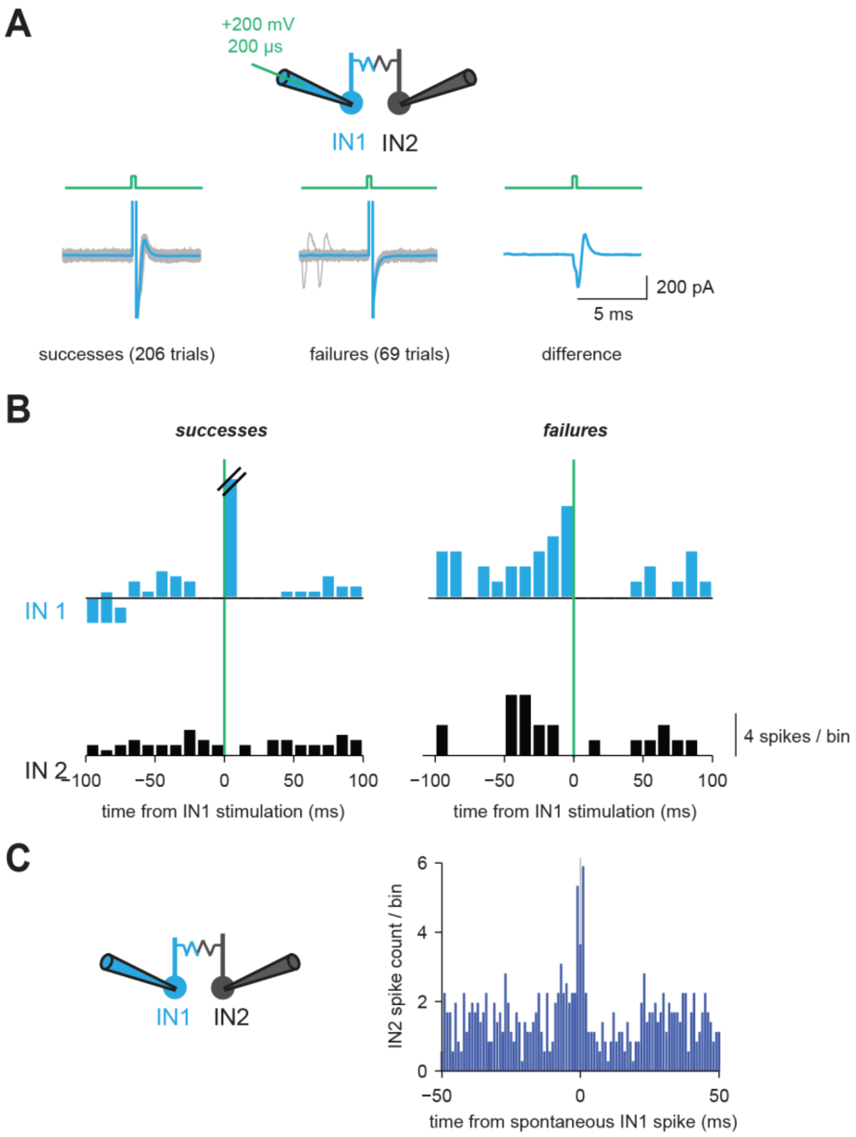

Figure S3. Triggered spikes in a single IN do not recruit a coupled IN. Related to Figures 1 and 2

(A) Top: Configuration of a dual IN recording. IN1 was stimulated with 200 mV, 200 µs voltage pulses. Below: Voltage pulses tiggered temporally precise single spikes in IN1, with the repolarizing spike phase obvious after stimulus offset. Left: Successful trials, middle: Failure trials, right: Mean waveform difference between success and failure trials. (B) Top: Histograms of spikes in IN1 in success trials (left) and failure trials (right). Below: Histograms of simultaneously recorded spikes in IN2. Note that spike induction in IN1 did not induce spikes in IN2. Bin size = 10 ms. Green lines indicate times of IN1 stimulation. (C) Left: Recording configuration. Right: Binned spike counts of IN2 aligned to spikes in IN1. Note the peaks at ± 1 ms, indicative of gap-junction coupling between IN1 and IN2.

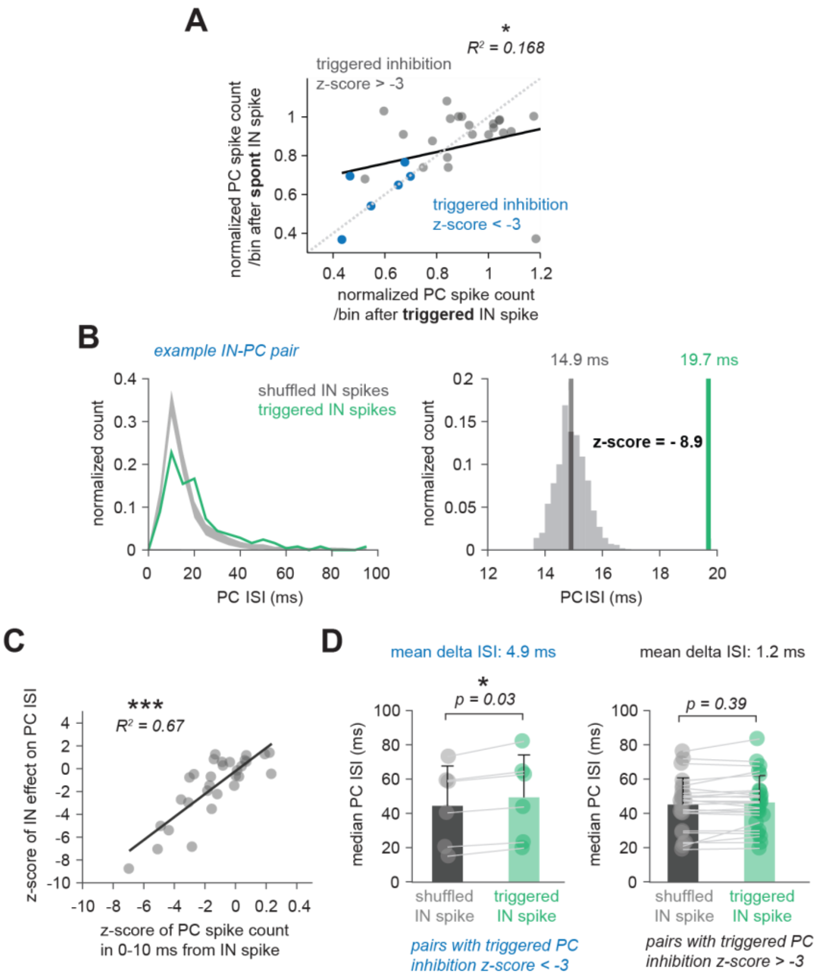

Figure S4. Quantification of IN-PC inhibition as effects on PC ISIs.

Related to Figure 1

(A) Baseline-normalized PC spike counts / bin after spontaneous and after triggered IN spikes are correlated (black: regression line, dotted: unity line). Note that pairs with significant PC inhibition after triggered spikes (z-score <-3, blue) are close to the unity line. (B) Left: In an example IN-PC pair, the distribution of PC ISIs around triggered IN spikes (green) and the mean ± SD of PC ISI distributions around shuffled IN spike times (grey, n = 1000 shuffles) are shown. Right: The median of the real distribution is compared to the distribution of medians from shuffled spike times and a z-score is calculated. (C) Z-scores of IN effects on PC ISIs correlate strongly with z-scores calculated from baseline-normalized PC spike counts. (D) Left: For pairs with significant IN-PC inhibition, PC ISIs around triggered spikes were on average 4.9 ms longer than PC ISIs around shuffled IN spike times. Right: For pairs without significant IN-PC inhibition, PC ISIs were not significantly different between real and shuffled IN spike times.

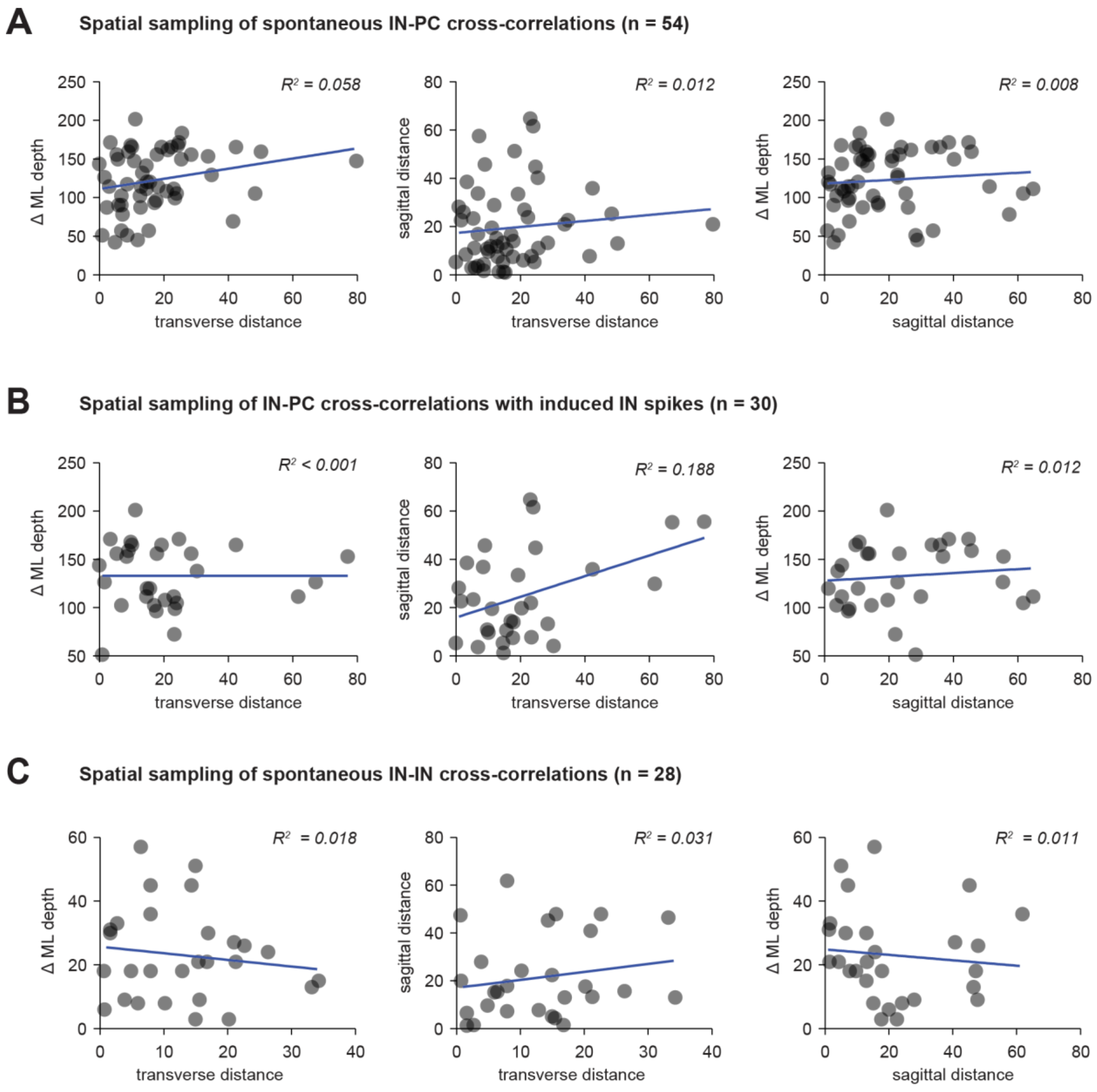

Figure S5. Spatial sampling of IN-PC and IN-IN pairs. Related to Figures 1, 2, 3

(A) Spatial sampling of spontaneous IN-PC cross-correlograms. Left: transverse versus molecular layer (ML) depth distances sampled. Middle: transverse versus sagittal distances sampled. Right: Sagittal versus ML depth sampled. Blue: Linear regression lines. Low R^2 values indicate that spatial sampling did not exhibit correlations between different planes. (B) Same as (A), but for spatial sampling of IN-PC pairs with induced IN spikes. (C) Same as (A, B), but for dual IN recordings.

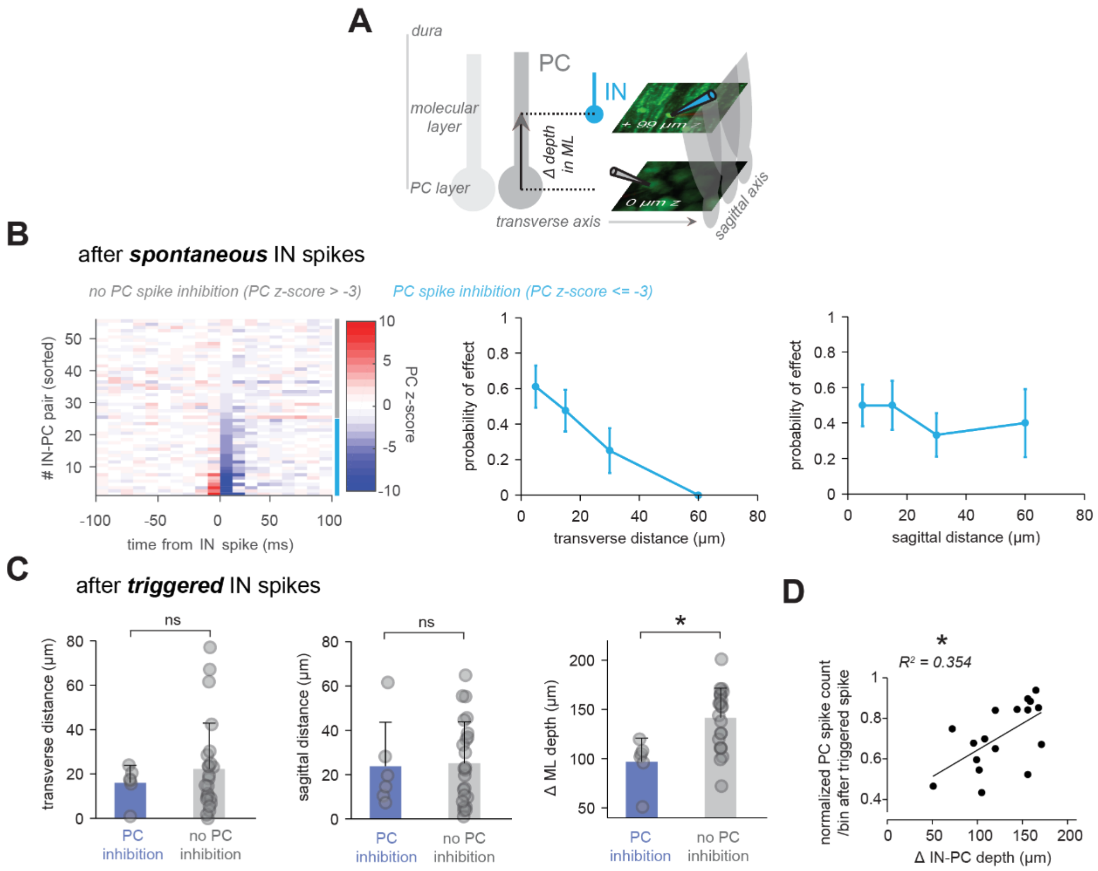

Figure S6. Distance-dependence of IN-PC inhibition. Related to Figure 1

(A) Illustration of the different spatial planes in which IN-PC distance was measured as intersomatic Euclidean distance. The center of the PC soma denotes 0 µm distance. (B) Left: For each IN-PC pair, z-scores of PC spiking aligned to spontaneous IN spikes are shown (color axis clipped at z-score = ±10 for better visualization, bin size = 10 ms). Pairs were sorted by minimal z-score in 0-10 ms bin. Light blue bar shows IN-PC pairs with significant PC inhibition (PC z-score < -3), grey bar shows pairs without significant PC inhibition (z-score > -3). Middle: Fraction of pairs with significant IN-PC inhibition across transverse IN-PC distance. Error bars show SD based on bootstrap analysis. Right: Same for sagittal distance. (C) Left: comparison of ML depth IN-PC distances for pairs with and pairs without significant PC inhibition after triggered IN spikes. Error bars denote SD. Middle: Same for sagittal IN-PC distance. Right: same for IN-PC depth distance in ML. (D) For IN-PC pairs within 30 µm intersomatic transverse distance, ML depth IN-PC distance is plotted versus the normalized PC spike count / bin 0-10 ms after the triggered IN spike. The black line indicates the regression line.

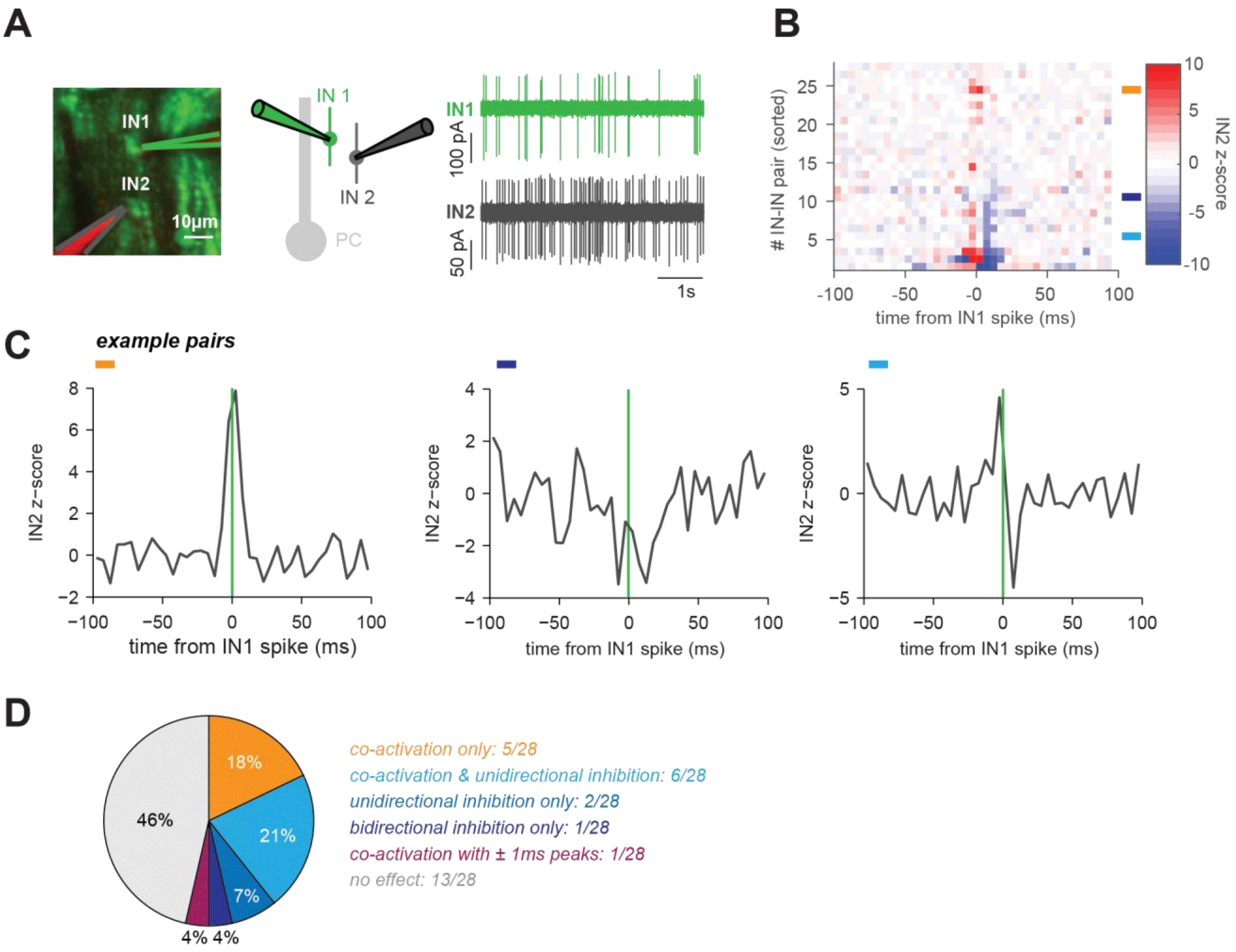

Figure S7. IN-IN cross-correlograms show various combinations of synchronization and inhibition. Related to Figure 2

(A) Left: Two-photon average intensity projection showing a simultaneous IN-IN cell-attached recording. Middle: Schematic of paired recording configuration. Right: Spontaneous raw activity of IN-IN pair. (B) For each IN-IN pair, z-scores of IN2 spiking aligned to IN1 spikes are shown (color axis clipped at z-score = ±10 for better visualization, bin size = 5 ms). Pairs were sorted by minimal z-score in 0-5 ms bin. IN1 and IN2 definition was flipped if the cross-correlogram suggested unidirectional inhibition from IN2 to IN1. (C) Examples of IN-IN cross-correlograms with different profiles (bin size = 5 ms). Colored bar in top left of panels indicate identity of those cross-correlograms in overview in B. (D) Relative frequencies of different types of IN-IN interactions across all IN-IN recordings, see Methods for categorization.

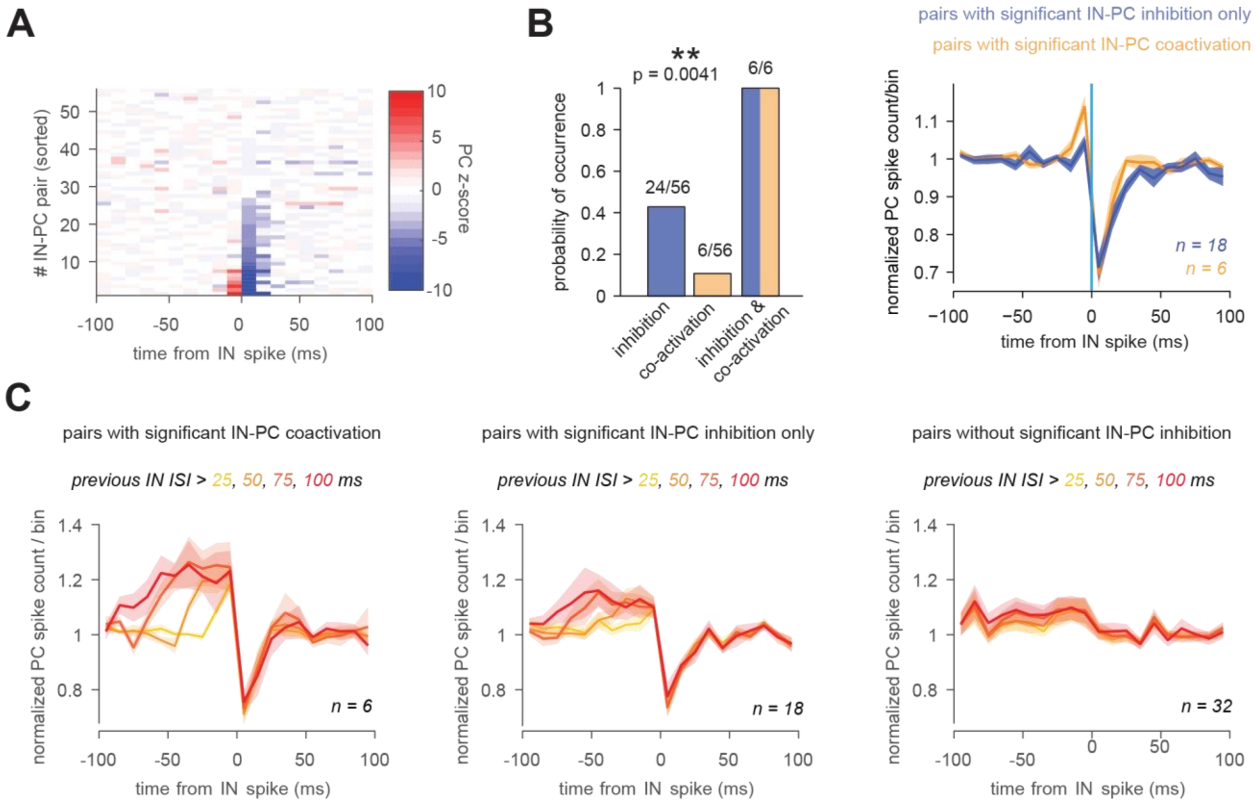

Figure S8. IN-PC co-activation before PC inhibition may stem from common granule cell input and / or PC disinhibition. Related to Figure 1

(A) For each IN-PC pair, z-scores of PC spiking aligned to IN spikes are shown (color axis clipped at z-score = ± 10 for better visualization, bin size = 10 ms). Pairs were sorted by minimal z-score in 0-10 ms bin. Same as in Fig 2B. (B) The probability of observing IN-PC inhibition (blue) is 100% in pairs with significant IN-PC co-activation (orange). The association between IN-PC inhibition and co-activation is significant (Fisher's two-tailed exact test). Right: Normalized PC spike counts / bin for pairs with significant IN-PC co-activation (orange) and pairs with significant IN-PC inhibition only (blue). Shadings denote SEM. Bin size = 10 ms. (C) IN spikes are categorized depending on the previous ISI duration (shortest minimum ISI: 25 ms (yellow), longest minimum ISI: 100 ms (red), increments of 25 ms. Average normalized PC spike counts / bin are plotted for the different IN ISI categories. Left: Average of pairs with significant IN-PC co-activation, middle: average of pairs with significant IN-PC inhibition only, right: average of pairs without significant IN-PC effects. Note that in the left and middle plots, increases in PC spike counts preceding IN-PC inhibition follow the duration of the previous IN ISI.

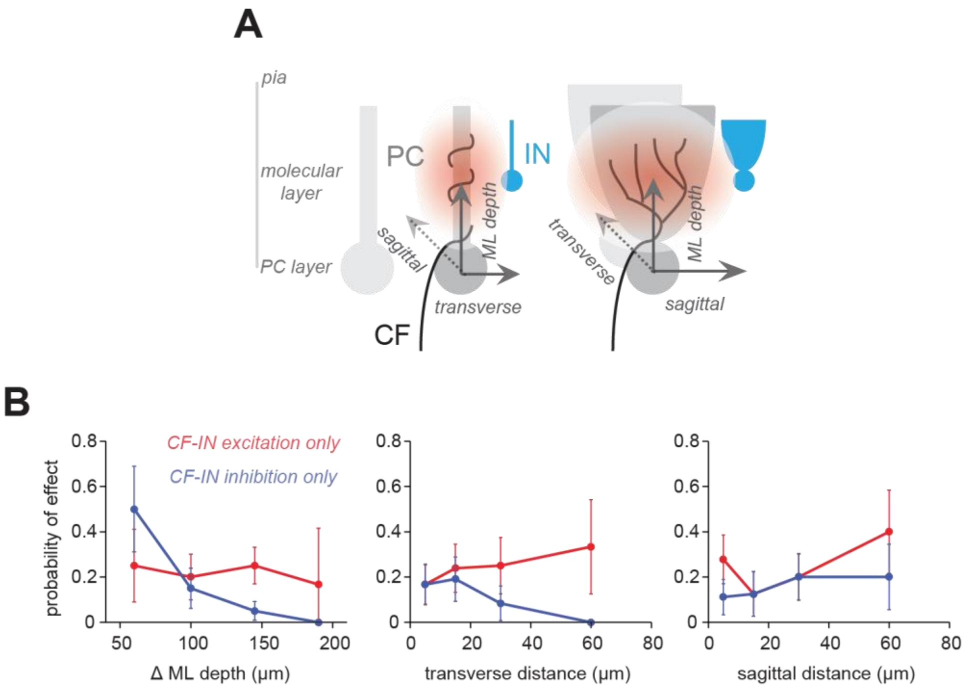

Figure S9. Distance-dependence of climbing fiber-IN effects. Related to Figure 3

(A) Illustration of the different spatial planes in which IN-PC distance was measured as intersomatic Euclidean distance. The center of the PC soma denotes 0 µm distance. (B) IN-PC pairs were categorized by IN spike responses to spontaneous CF input: significant CF-IN excitation only (red) and significant CF-IN inhibition only (blue). Fraction of pairs plotted across molecular layer depth (left, same as in Fig 4E), transverse (middle) and sagittal (right) distance. Error bars show SD based on bootstrap analysis.
